# Structural characterisation of molecular conformation and the incorporation of adatoms in an on-surface Ullmann-type reaction

**DOI:** 10.1038/s42004-020-00402-0

**Published:** 2020-11-11

**Authors:** Chris J. Judd, Filipe L. Q. Junqueira, Sarah L. Haddow, Neil R. Champness, David A. Duncan, Robert G. Jones, Alex Saywell

**Affiliations:** 1 School of Physics & Astronomy, The University of Nottingham, Nottingham, NG7 2RD UK; 2 School of Chemistry, The University of Nottingham, Nottingham, NG7 2RD UK; 3 Diamond Light Source, Harwell Science and Innovation Campus, Didcot, OX11 0QX UK

**Keywords:** Surface spectroscopy, Characterization and analytical techniques, Two-dimensional materials, Scanning probe microscopy

## Abstract

The on-surface synthesis of covalently bonded materials differs from solution-phase synthesis in several respects. The transition from a three-dimensional reaction volume to quasi-two-dimensional confinement, as is the case for on-surface synthesis, has the potential to facilitate alternative reaction pathways to those available in solution. Ullmann-type reactions, where the surface plays a role in the coupling of aryl-halide functionalised species, has been shown to facilitate extended one- and two-dimensional structures. Here we employ a combination of scanning tunnelling microscopy (STM), X-ray photoelectron spectroscopy (XPS) and X-ray standing wave (XSW) analysis to perform a chemical and structural characterisation of the Ullmann-type coupling of two iodine functionalised species on a Ag(111) surface held under ultra-high vacuum (UHV) conditions. Our results allow characterisation of molecular conformations and adsorption geometries within an on-surface reaction and provide insight into the incorporation of metal adatoms within the intermediate structures of the reaction.

## Abstract (web summary)

Ullmann-type reactions on metal surfaces are widely studied examples of on-surface synthesis. Here the combination of normal incidence X-ray standing wave analysis, X-ray photoelectron spectroscopy, and scanning tunneling microscopy enables the characterisation of molecular conformations in two such reactions.

## Introduction

The on-surface synthesis of covalently coupled molecular architectures is a promising route towards functional nanoscale materials^1^. Building upon approaches developed within solution phase synthetic chemistry, various on-surface synthesis protocols have been employed to produce extended 1D and 2D molecular structures; including nanoribbons^2^, porphyrin-based polymers^3,4^, and other structures^5–7^. These studies are often performed on metallic substrates held under ultrahigh vacuum (UHV) conditions and are predominantly based upon Ullmann-type and Glaser-type coupling, although other variants have been used, and common within these methodologies is the use of the substrate to drive on-surface bond-breaking reactions (reviewed in refs. ^8–12^).

A consequence of confining the reactions to a substrate is the transition from the “three-dimensional” reaction volume of solution phase synthesis to a quasi-two-dimensional platform. This new environment has the potential to facilitate alternative reaction pathways to those available in solution. An example of this, in the case of Glaser-type coupling, is the use the steric hindrance induced from 2D confinement to reduce branching reactions in the synthesis of linear structures^13,14^. To fully utilise this alternative environment a detailed understanding of the underlying mechanistic processes underpinning these reactions is required. The role of molecular conformation for surface adsorbed species has previously been observed to play a role in the progression of on-surface reactions^2,15^, and an additional consideration is the precise role of the metal surface (e.g., adatoms and surface defects) which have been observed to play a role in on-surface reactions.

Recently various methodologies have been developed to influence and control on-surface reactions. Native surface structures (for example, step-edges^16,17^ and the atomic corrugation of the substrate plane^18^) have been used to align the reactants, intermediates, and products of surface confined reactions. Building upon these concepts has facilitated the use of surface “templating” by utilising surface reconstructions^19^, porous molecular assemblies^20^, and large cyclic polymers^21^ as a way to direct the progress of these on-surface reactions.

Scanning tunnelling microscopy (STM) has been the predominant characterisation technique for such systems. The sub-angstrom lateral resolution and real-space imaging abilities has facilitated study of the initial, intermediate, and final states of on-surface reactions, even down to the level of individual bond formation in the case of the related noncontact atomic force microscopy (ncAFM) technique^22–24^. However, the family of scanning probe microscopy (SPM) techniques lack detailed chemical specificity, compared to X-ray photoelectron spectroscopy (XPS)-based techniques, and do not facilitate the accurate characterisation in terms of molecular adsorption heights^25^. Specifically, accurate structural characterisation of adsorption geometry and molecular conformations is required to provide information on details of the underlying reaction mechanism.

Studies of Ullmann-type coupling utilising XPS have provided information on the chemistry of the molecular structures at various stages of the reaction^26^, and time-resolved XPS studies have allowed the reaction kinetics of an on-surface process to be probed^27,28^. These studies provide useful information, but questions remain as to the evolution of the conformation of the molecular species during the reaction and the role of surface adatoms (of particular importance for Ullmann-type reactions studied within this work).

Here, we demonstrate that by combining the exceptional lateral resolution of STM with the chemical sensitivity of X-ray standing wave (XSW) structure determination using photoelectron detection^29,30^, it is possible to characterise the molecular conformations and adsorption geometries within an on-surface reaction covalent coupling reaction. In addition we are able to provide insight into the incorporation of metal within the intermediate structures as part of the reaction mechanism, evidenced by the structure of the intermediate state of the Ullmann coupling reaction of two molecular species on Ag(111).

## Results

**Fig. 1:**
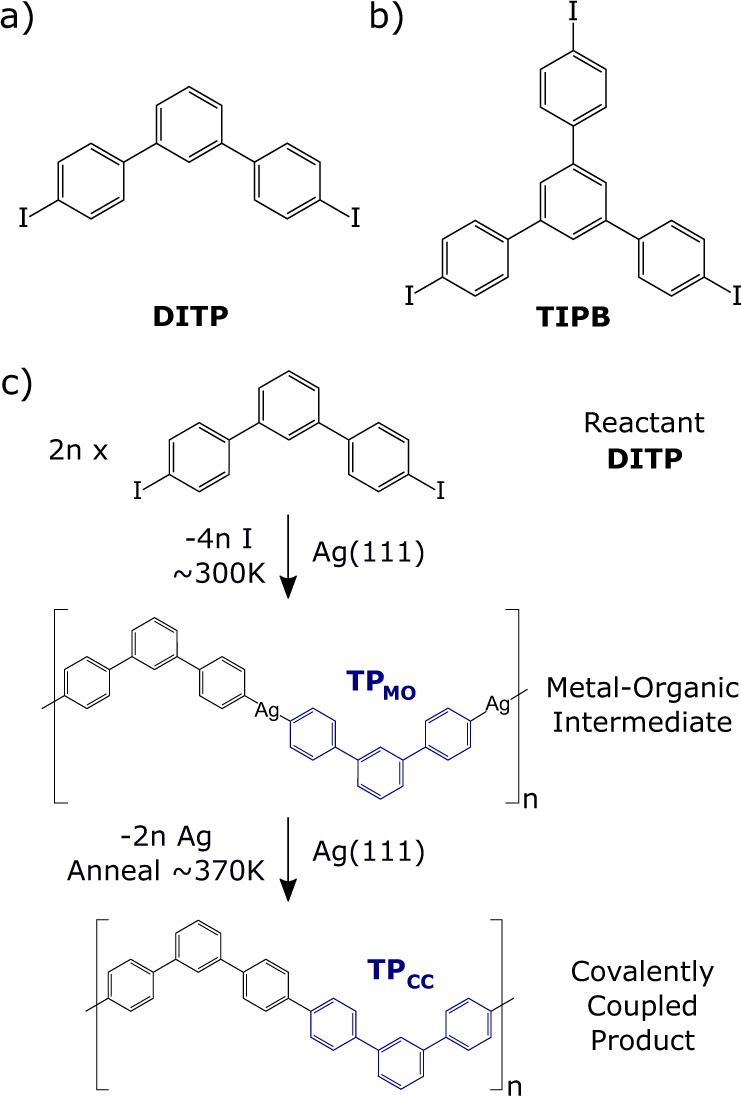
Chemical structure of reactant molecules studied and scheme showing the progression of an Ullmann-type on-surface reaction. Chemical structures of **a** 4,4″-diiodo-m-terphenyl (DITP) and **b** 1,3,5-tris(4-iodophenyl)benzene (TIPB). **c** Reaction scheme for DITP on Ag(111) where cleavage of the C–I bond and formation of an extended metal-organic intermediate, TP_MO_, occurs at 300 K (room temperature). Annealing at 370 ± 50 K, based on XPS data, results in the formation of a covalently coupled product, TP_CC_ (see refs. ^18,20^).

Ullmann-type on-surface coupling reactions result in the formation of covalent products and progress via a metal-organic intermediate (typically with a thermally activated reductive elimination step required to remove the metal atom from the complex facilitating the coupling of the organic units). Our study presents the characterisation of two molecules, 1,3,5-tris(4-iodophenyl)benzene (TIPB) and 4,4″-diiodo-m-terphenyl (DITP), both of which possess aryl rings functionalised at the periphery with C–I groups to facilitate Ullmann coupling reactions when deposited upon a Ag(111) substrate (chemical structures shown in Fig. 1a, b).

As in our previous studies of DITP^18^ and TIPB^20^ on Ag(111) it is expected that covalent coupling on Ag(111) progresses via a metal-organic intermediate; Fig. 1c shows the reaction scheme for DITP on the Ag(111) surface. Adsorption of DITP and TIPB onto a Ag(111) substrate held at room temperature results in the cleavage of the C–I bond and the formation of a metal-organic (MO) structure. The dehalogenated molecular species are connected via organometallic (C–Ag–C bonds) within the MO structure. We denote the subunits within these structures as m-terphenyl (TP_MO_) and 1,3,5-triphenylbenzene (TPB_MO_)—indicating the relationship to the reactant molecules DITP and TIPB, respectively. Following the formation of the MO structure the provision of additional thermal energy gives rise to formation of the covalently coupled (CC) product (370 K and 400 K to produce TP_CC_ and TPB_CC_, respectively).

**Fig. 2:**
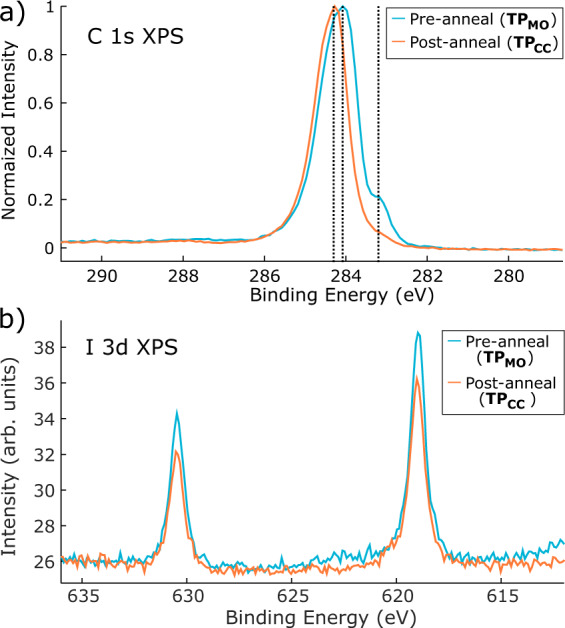
C 1s and I 3d photoelectron spectra acquired for the metal-organic and covalent phases of TP on Ag(111). The metal-organic phase (TP_MO_) is present on the surface following deposition, and the covalent phase (TP_CC_) is formed following annealing at 370 ± 50 K. **a** C 1s photoelectron spectra, obtained using a photon energy of 435 eV, before (blue) and after (orange) annealing. **b** I 3d photoelectron spectra, obtained using a photon energy of 770 eV on Ag(111) before (blue) and after (orange) annealing.

Initial characterisation of the on-surface reaction was conducted using XPS measurements to identify the distinct chemical species present within the MO and covalently-coupled structures. Figure 2a shows the C 1s photoelectron spectra obtained on Ag(111) for TP_MO_ (i.e. in the MO phase; preanneal) and TP_CC_ (covalent phase; postanneal). For the MO phase two features are identified. In agreement with previous work, we assign the major peak (284.1 eV) to aromatic carbon species within the backbone of TP_MO_, with the shoulder at lower BE (283.2 eV) attributed to organometallic C–Ag–C species within the MO phase (NB we assign the absence of features at higher binding energies to the lack of intact aryl-halides species i.e., nondissociated C–I bonds)^28,31^.

Following annealing the surface to 370 ± 50 K, a temperature at which initiation of the on-surface covalent coupling is expected, the main peak (284.3 eV) is still present and a substantial attenuation in the relative intensity of the shoulder feature is observed (normalised intensities are shown in Fig. 2a). These findings are consistent with conversion of the majority of the molecular species present to extended covalent structures (i.e., reductive elimination of the Ag from the organometallic C–Ag–C bond to give rise to a new C–C bond, resulting in reduction in intensity of the shoulder peak). The slight shift, by 0.2 eV, of the main peak towards higher binding energy is in agreement with several similar results for covalent coupling on surfaces; previously attributed to differences in charge transfer between the surface and metal-organic and covalent phases^27,32^, although effects such as core-hole screening may also play a role. Details of the C1s XPS data for TPB_MO_ and TPB_CC_ are included in the Supporting Information, Fig. S1.

The I 3d photoelectron spectra for TP within MO and covalent phases on Ag(111), shown in Fig. 2b, support our reaction model where cleavage of the C–I bond, and formation of Ag–I with the substrate, occurs following deposition onto the surface at ~300 K. Two peaks are present in the spectra, assigned to I 3*d*_3/2_ (630.5 eV) and I 3*d*_5/2_ (619.0 eV). Both prior to, and after, annealing to 370 K a single chemical environment for iodine is observed (as evidenced by the constant BE). The I 3*d*_5/2_ peak is at a binding energy consistent with chemisorbed iodine bonded to Ag (*c.f*. chemisorbed I–Ag formed by the dissociative chemisorption of methyl iodide at a clean Ag(111) surface at 290 K^33^), in agreement with our assertion that the C–I bond within DITP has dissociated following deposition. Similar results are observed for TIPB (see Fig. S2 in the Supporting Information).

### Molecular adsorption and conformation: chemically sensitive normal incidence X-ray standing wave (NIXSW) analysis

The identification of specific chemical states via XPS characterisation of the intermediate and final structures of the on-surface reaction facilitates our use of chemically sensitive NIXSW studies to perform structural characterisation^29,30^. NIXSW analysis based upon the specific chemical environments defined above (e.g., aromatic and organometallic carbon species) provides two structural parameters; a coherent fraction (*C*_f_) and a coherent position (*C*_p_). Here, we focus on the C 1s signatures for the aromatic and organometallic carbon species (as characterised above) to determine the coherent fraction and position of the species relative to the (111) and (200) crystal planes of the bulk Ag substrate.

**Fig. 3:**
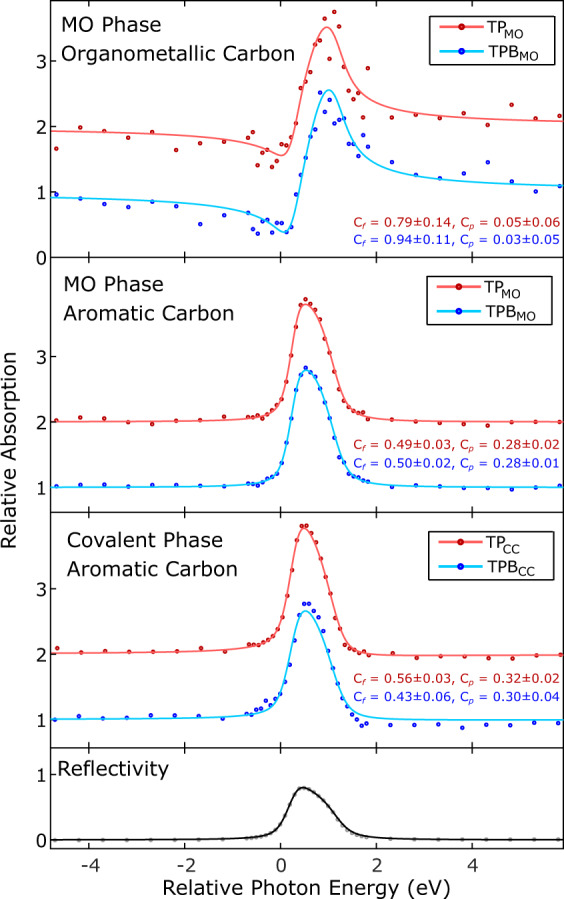
Chemically sensitive NIXSW measurements of the C1s region of TP and TPB within the metal-organic (MO) and covalently coupled (CC) phases. NIXSW photoelectron yields obtained using the (111) reflection of the Ag substrate for the C 1s core level, comparing MO and covalent phases of TP (red) and TPB (blue). Each profile is normalised to 1 away from the Bragg condition, with TP results offset by 1 unit for clarity. The obtained *C*_f_ and *C*_p_ values are shown for each fit.

Figure 3a shows the NIXSW absorption yields of C 1s peaks, obtained from the (111) reflection from the Ag substrate, for TP and TPB (details of fit in Figs. S3 and S4). We focus initially on the organometallic carbon atoms forming a bond with a silver species (C–Ag–C). The profiles obtained from the XSW measurements (Fig. 3—top) show a similar form for both TPB_MO_ and TP_MO_. The TPB_MO_ measurements yield a coherent fraction of *C*_f(111)-C1s_ = 0.94 ± 0.11. This high coherent fraction suggests a high degree of order and most likely indicates a single adsorption site for all organometallic carbon species within the TPB_MO_ upon the Ag(111) surface (a slightly lower level of order is found for TP_MO_, *C*_f(111)-C1s_ = 0.79 ± 0.14). The coherent position values, which are given as fractions of the (111) layer spacing, for the organometallic carbons of both molecules agree within error (*C*_p(111)-C1s_ = 0.05 ± 0.06 and *C*_p(111)-C1s_ = 0.03 ± 0.05 for TP_MO_ and TPB_MO_, respectively). This indicates that the C species forming the C–Ag–C bond in both molecules are adsorbed at similar heights above the surface (0.25 ± 0.01 nm and 0.24 ± 0.01 nm for TP_MO_ and TPB_MO_, respectively). The agreement in adsorption height for these two related but structurally different species show that the observations offer insight into a more general feature of molecule-surface interactions for the intermediates of Ullmann-type on-surface coupling.

It should be noted that all obtained XSW distances are relative to the extended crystallographic planes of the bulk Ag crystal. Therefore the adsorption “heights” relative to the surface of the Ag(111) crystal are distances are from the outermost (111) plane. We base our analysis on the assumption that the surface Ag atoms reside on this plane, thereby allowing the construction of adsorption models.

XSW profiles obtained from analysis of the aromatic carbon species in both MO and covalent structures are shown in Fig. 3—middle. These profiles show a high degree of similarity, with fits to all profiles resulting in similar coherent fraction and position values. Notably, the coherent fraction values for the aromatic carbon species are significantly smaller than 1 (~0.5 for TP and TPB in both the MO and covalent phases), indicating that the aromatic carbon atoms within the molecules are adsorbed at multiple heights.

**Fig. 4:**
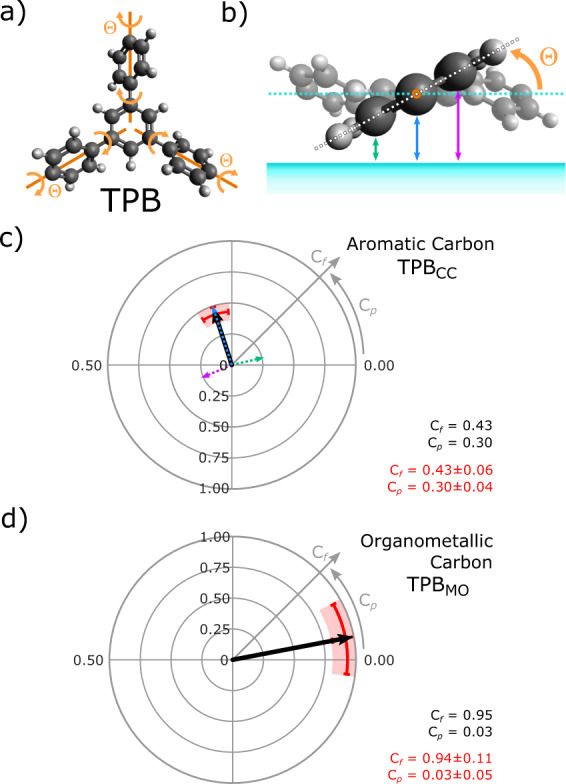
Structural model for the conformation of TPB_CC_ on Ag(111). **a** Model showing twisting of TPB_CC_ aryl end groups around axes of rotation (orange) parallel to the (111) plane and the plane of the molecule; Θ—dihedral angle. **b** Side view of twisted TPB_CC_ model. Arrows show three different heights of carbon atoms above the surface: above (purple), below (green) and level with (blue) the molecular plane (dashed cyan line). **c** Argand diagram showing *C*_f_ and *C*_p_ values for aromatic carbon atoms in TPB_CC_. XSW results (red) with a model for twisted TPB_CC_ (Θ = 30°) overlaid. Dashed arrows show vectors for carbon atoms at the three different heights above the surface (colours match those in **b**), with the resultant vector shown in black. **d** Argand diagram showing model of organometallic carbon atoms in TPB_MO_, based on measurements of *C*_f_ and *C*_p_. Both argand diagrams show the resultant vector *C*_f_ and *C*_p_ values in bottom right.

Although both the MO intermediate phase and covalently coupled final reaction products are extended structures we are able to analyse the structure of the monomer units within the 2D networks. Our interpretation is a model where the outer aryl groups, i.e., those with para connections, of each molecule (monomer unit) are twisted relative to the central ring, i.e. those with meta connections (see Fig. 4a). The central aryl groups are locked in place (due to the meta connections) and are expected to be parallel to the substrate surface. The para groups can twist, and this twisting results in the aromatic carbon atoms within the molecule being orientated at several different heights above the surface. To model the structure it is assumed that the central aryl ring, and carbon atoms on the rotational axes of the terminal aryl rings, remain flat relative to the surface in a single plane (i.e., the molecular plane is parallel to the (111) plane of the surface), and that all terminal aryl groups are rotated by the same dihedral angle, Θ (example for TPB_CC_ is shown in Fig. 4b—additional details of the model are presented in Supplementary Figs S5 and S6 and associated text).

Calculated values for *C*_f_ and *C*_p_ based upon this model for TPB_CC_, with Θ = 30°, are presented on an Argand diagram (Fig. 4c—each arrow is a vector showing *C*_f_ and *C*_p_ values in polar co-ordinates). Dashed arrows represent aromatic carbon atoms higher (purple), lower (green), and level with the molecular plane (blue). The solid black arrow shows the predicted *C*_f_ and *C*_p_ values for our model and is in excellent agreement with experimental values (red region indicates data point and estimate of corresponding error).

From the experimental values of *C*_f_ and *C*_p_ for the aromatic carbon species we are able to calculate Θ for MO and covalent phases of both molecules. These results (shown in Table S1) show the side aryl groups in all cases are twisted by approximately 30° (30 ± 3° for TPB_CC_). For comparison, biphenyl molecules in free space have a twist angle of 44° (due to steric hindrance between the hydrogens on adjacent phenyl groups)^34^. The observation that the dihedral angle is reduced for the surface-confined molecule compared to a molecule in free space can be understood due to the interaction with the surface; flattening of the molecule maximises the molecule–surface interaction with the π orbitals of the terminal aryl rings. We emphasise that the use of structural characterisation via NIXSW in concert with STM can provide information about the progression of on-surface reactions. Our findings here support the previously suggested conformation flexibility of intermediates with Ullmann-type on-surface reactions^2,15^.

### Incorporation of metal adatoms within MO structures

**Fig. 5:**
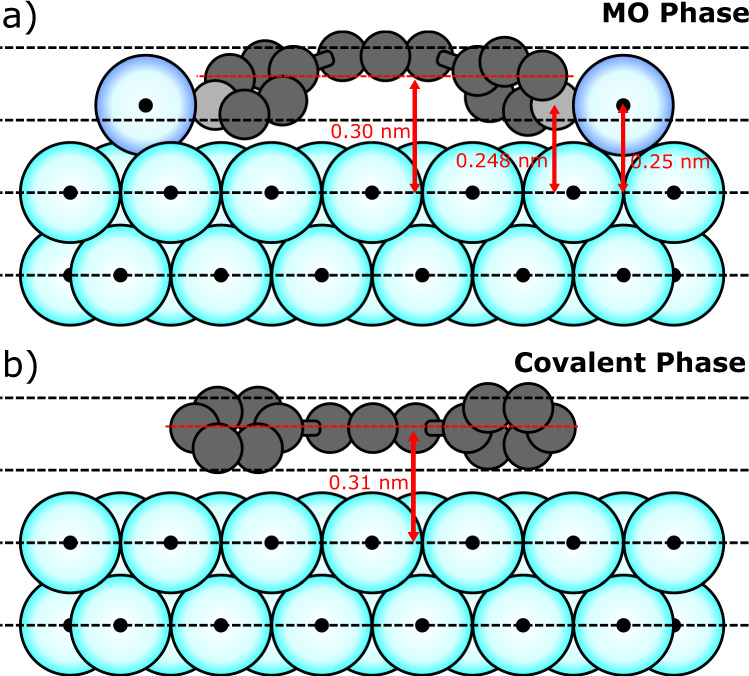
Models for the vertical positioning of molecules above the Ag(111) surface. **a** Metal-organic intermediate phase of the Ullmann-type coupling reaction, with molecules bonded to silver adatoms (dark blue) adsorbed in bridge sites. Average heights of the molecule (red dashed line), C–Ag–C bonded carbon (light grey) and Ag adatoms are shown. **b** Covalent phase of Ullmann coupling reaction, with average height of the molecule shown.

We now address an important consideration relating to the bonding of the organometallic carbon species within the MO phase. The organometallic carbon atoms are found to be 0.25 ± 0.01 nm and 0.24 ± 0.01 nm above the surface, for TP_MO_ and TPB_MO_, respectively (Fig. 4d shows an argand diagram of the results for coherent fraction and position values for organometallic carbons). This can be compared to the average heights of the aromatic carbons within the MO phase; both TPB_MO_ and TP_MO_ are found to be ~0.30 nm relative to the height of the topmost Ag(111) plane (0.302 ± 0.005 nm and 0.302 ± 0.002 nm above the surface, for TP_MO_ and TPB_MO_, respectively), in agreement with results for relative heights above surfaces for large π-conjugated molecules on Ag(111) (0.29 nm for diindenoperylene adsorbs on Ag(111)^35^). The value of the adsorption height is significantly lower for organometallic carbon species than the purely aromatic, resulting in a curved structure for the adsorbed molecular species (see Fig. 5a). This indicates that the end aryl groups are likely to bend down towards the surface when forming the organometallic C–Ag–C bond.

Previous computational studies of Ullmann-type on-surface coupling reactions, employing density functional theory (DFT), have suggested that MO structures are bound to substrate lattice atoms^36^. This is in contrast to experimental findings where structures are characterised as incorporating surface adatoms^15,26,28,37^. Our NIXSW analysis indicates that the organometallic carbon atoms are significantly higher above the surface (~0.25 nm) than a single C–Ag bond length (approximately 0.21 nm^38^), implying that the Ag species involved in the metal-organic bond is unlikely to be in the surface silver layer. The metal-organic bond must therefore be made with either a surface adatom or a silver atom that has been significantly raised out of the surface. Our STM data for the same molecule-substrate systems do not provide evidence for removal of Ag atoms from surface layer (likely to result in the presence of etch pits which are not observed in this study, although monolayer vacancies have been observed for haloarenes on metal surfaces^39^), we therefore suggest that Ag adatoms diffusing from low-coordinated step-edge sites play a significant role. Our proposed model for TP in the metal-organic phase of the Ullmann-coupling reaction (TP_MO_), including the labelled heights of the organometallic carbon atoms, is shown in Fig. 5a. The adsorption height of the organometallic carbons are compatible with Ag adatoms located at bridge or 3-fold hollow sites (further details of the adsorption geometry are given below).

**Fig. 6:**
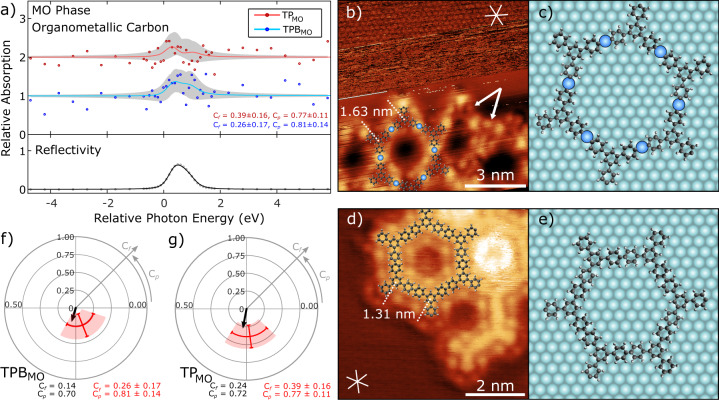
Structural characterisation of the adsorption geometry for the metal-organic (MO) and covalently coupled (CC) phases of TPB on Ag(111). **a** NIXSW photoelectron yields obtained using the (200) reflection for the C 1s core level, comparing TP_MO_ (red) and TPB_MO_ (blue). Grey shading shows curves fitted using values of *C*_f_ and *C*_p_ within quoted uncertainty range. Each profile is normalised to 1, away from the Bragg condition, with TP_MO_ results offset by 1 unit for clarity. The obtained *C*_f_ and *C*_p_ values are shown for each profile. **b** STM image showing the metal-organic phase of TPB. Top section shows a region of atomic contrast, used to calibrate image. Molecular model, intermolecular separations and lattice vector directions are overlaid. White arrows indicate example locations of iodine atoms. Imaging parameters: *V*_bias_ = −80mV, *I*_set_ = 2 nA for the top section, *V*_bias_ = −1.8 V, *I*_set_ = 20 pA for bottom section. **c** Proposed adsorption models for metal-organic phases of TPB on Ag(111) based on XSW modelling. **d** STM image of covalently bonded TPB on Ag(111) after annealing the surface. Molecular model, intermolecular separations and lattice vector directions are overlaid. Image parameters: *V*_bias_ = −1.8 V, *I*_set_ = 20 pA. **e** Proposed adsorption model for covalent phase of TPB. Models based on measurements obtained from STM images. **f**, **g** Argand diagrams for TPB_MO_ and TP_MO_ respectively, showing resultant vector (black) for organometallic carbon atom locations relative to the (200) plane, based on proposed adsorption models (**c**, **e**). Measured coherent fraction and position values are shown in red and the model *C*_f_ and *C*_p_ values are given in the bottom left.

Following completion of the reaction the covalent phases of TP and TPB (now consisting exclusively of aromatic carbon species) were both found to be adsorbed at an average of 0.31 ± 0.01 nm above the surface, with the end aryl groups twisted around a central axis (as discussed above). A model for a single TP_CC_ molecule in the covalent phase is shown in Fig. 5b (plan view of adsorption shown in Fig. 6e—and discussed below).

### Structural characterisation: adsorption geometry

Our characterisation facilitates the determination of adsorption sites for the organometallic carbon species via a “triangulation” process^30^. NIXSW measurements from the (111) and (200) reflection planes of the Ag(111) are used to provide information on the adsorption sites for specific chemical species. For example, by using the I 3d_5/2_ XPS signal and both the (111) and (200) reflections the structure of the co-adsorbed iodine has been completely determined; with iodine adsorbed at three-fold hollow sites in a 1:1 ratio of face centred cubic and hexagonally close-packed three-fold hollow sites (fcc:hcp—See Fig. S7 and Table S2). The adsorption geometries of organometallic carbon atoms within both TP_MO_ and TPB_MO_ can be determined in a similar manner.

XSW profiles obtained using the (200) reflection of the Ag(111) surface provide information on the organometallic carbon atoms within the TP_MO_ and TPB_MO_ structures (Fig. 6a—*C*_f_ and *C*_p_ values obtained from the fitted curve are shown, see SI for details of fitting). The data for the organometallic species results in low values for the coherent fractions (*C*_f(200)-C1s_ = 0.39 ± 0.16 and *C*_f(200)-C1s_ = 0.26 ± 0.17 for TP_MO_ and TPB_MO_, respectively) from which we may infer that the organometallic carbon atoms are adsorbed at multiple positions relative to the (200), excluding sole adsorption in atop, fcc, or hcp hollow sites.

In order to facilitate an interpretation of the XSW measurements and to produce an adsorption model of the MO and covalently bonded TPB and TP structures we have also characterised the molecular species on the Ag(111) surface using STM. Figure 6b shows the edge of a MO structure formed from the as-deposited TIPB adsorbed on a Ag(111) surface (a model showing the position of TPB_MO_ molecules and Ag adatoms is overlaid). Individual TPB_MO_ units can be seen, connected by circular bright features (XPS results above demonstrate C–I cleavage has occurred following deposition). These bright features are therefore attributed to silver adatoms (similar to features previously observed for MO structures^26,37^). The molecular units exist as part of an extended porous structure, comprising mostly of hexagonal pores with some disordered regions of other polygonal arrangements (as previously observed for structures formed from TIPB^20^ and the brominated analogue^6^). Additionally, further bright features are observed adjacent to the molecules (examples indicated by white arrows in Fig. 6b) and are attributed to iodine atoms adsorbed to the surface after dissociating from the molecules (similar to that observed for studies on other iodinated species^15^).

Details of registry with the underlying surface can be obtained by imaging the underlying Ag(111) substrate (top region of Fig. 6b shows atomic resolution). Combined imaging of the substrate and molecular overlayer is obtained by pausing image acquisition at the edge of the island (following imaging of the molecular structure), and altering the bias voltage and current set-point. The region of atomic resolution was then used to calibrate the image in order to determine accurate distances, and angles of molecules relative to the surface crystallographic directions. Combining this with measurements from additional images of the same surface, the centre-centre separation for TPB units within the MO structure is found to be 1.63 ± 0.1 nm. This is consistent with the expected dimensions for two TPB molecules bridged by a C–Ag–C link (compare with 1.61 ± 0.1 nm from our previous studies of TPB_MO_^20^ and similar molecules, 1.61 ± 0.2 nm^26^). The axis of this bond is misaligned to the <$\bar 110$> set of crystal directions (substrate close-packed directions) by 25 ± 2°.

STM experiments were also performed on annealed surfaces for both TPB_CC_ and TP_CC_ to gain insights into their adsorption geometries. Figure 6d shows an area of TPB_CC_ forming covalent hexagonal structures on the surface following anneal (see Fig. S10 for overview of structure). Unlike the preanneal surface, molecules appear to have a constant apparent height along their structures with no features associated with Ag adatoms, indicating that the molecules are covalently bonded. Bright circular features are also seen surrounding TPB units and within hexagonal pores and are attributed to iodine. A model showing the molecular structure is overlaid. TPB units were found to be separated by 1.31 ± 0.03 nm (again in good agreement with previous work^6^), and aligned at an angle of 3 ± 2° to the surface <$\bar 110$> crystallographic directions (NB the rotation of the molecular lattice allows periodicity with the substrate to be maintained, as discussed in our previous work^18^).

Utilising a combination of the XSW measurement from the (111) and (200) planes in concert with the STM measurements described above a simplified model was developed to locate the molecular adlayer relative to the surface substrate layer. The model considers only the organometallic carbon atoms of molecules both for simplicity (and due to the low coherent fraction values obtained from the aromatic carbon data—see Figs. S8, S9 and Table S3 for (200) XSW data). From these atoms, the lateral position of the rest of the molecule can be inferred (full details for how the model is constructed are outlined in Fig. S11).

STM measurements of molecular separation and angle relative to the surface were used as starting values for variables in the model and allowed to vary within a small range. Additionally, the height of molecules above the surface was varied within a range of values determined from XSW measurements from the (111) reflection. Finally, the lateral position of molecules was varied with molecule positions limited such that the central aryl ring of all molecules was adsorbed in a high symmetry site (atop, bridge, or three-fold hollow). Coherent fraction and position values calculated for each model were compared to XSW measurements with the assumption that Ag adatoms were adsorbed in high symmetry sites. Models with acceptable values of *C*_f_ and *C*_p_ values were compared to STM measurements.

For both TPB_MO_ and TP_MO_, a single high symmetry structural arrangement was found in agreement with experiments (minor deviations from the high-symmetry sites fit within the error range of our experimental results). The adsorption model for metal-organic TPB_MO_ (Fig. 6c) shows all molecular centres in three-fold hollow adsorption sites (i.e., either (i) all hcp or (ii) all fcc sites), with Ag adatoms predicted to rest in the same three-fold hollow sites as the molecular centres (i.e., all (i) hcp or (ii) fcc). Lateral translation of the molecular overlayer, such that molecule centres are adsorbed at bridge sites (with adatoms positioned in bridging sites), also results in a model that agrees well with the STM and XSW results. Of note is that adsorption models with molecular centres adsorbed above atop sites, and hence Ag adatoms in atop sites, gave calculated values for *C*_f_ and *C*_p_ incompatible with the experimental values, and thus we can exclude such adsorption sites (this may be rationalised by considering the lower stability of Ag adatoms adsorbed in atop sites for this model).

The proposed adsorption model has a molecular centre to centre separation of 1.53 nm, in agreement with STM measurements (1.63 ± 0.1 nm). Additionally, the adsorption model predicts molecules are aligned to the high symmetry directions of the surface at an angle of 19° (deviation from the measured angle, 25 ± 2°, may be due to the nonuniform nature of the extended molecular structures which result from the inclusion of nonhexagonal pores). Argand diagrams showing the resultant *C*_f_ and *C*_p_ values for these models are shown in Fig. 6f, g. Based upon our model of the metal-organic phase of TPB we expect *C*_f(200)-C1s_ = 0.14 ± 0.04 and *C*_p(200)-C1s_ = 0.70 ± 0.03 (the “ranges” quoted account for variation in molecular flexibility which gives rise to several models which would fit with the experimental data—minor variations in height of the organometallic carbon atoms alter *C*_f(200)-C1s_ and *C*_p(200)-C1s_ while keeping the same high-symmetry adsorption site of the Ag adatoms and molecular centres). These values are in agreement with the experimentally determined XSW values (*C*_f(200)-C1s_ = 0.26 ± 0.17, *C*_p(200)-C1s_ = 0.81 ± 0.14). Details of the MO phase of TP are presented in the SI (Fig. S12).

## Conclusion

By employing a combination of XPS, STM, and chemically sensitive NIXSW measurements to characterise an on-surface Ullmann-type reaction we are able to determine the molecular conformation and adsorption geometries for intermediate (MO) and final (covalently bonded) phases. Structural characterisation of the adsorption geometries for organometallic carbons, within the MO intermediate phase, indicates that it is metal adatoms which are incorporated within the intermediate complex (and not atoms within the surface layer). It is therefore likely that strategies seeking to control on-surface reactions based upon limiting the availability of such species are a promising route towards bespoke functional materials.

This approach of utilising the exceptionally high-lateral resolution of STM alongside the chemical sensitivity and vertical resolution (relative to the plane of the substrate) of NIXSW underpins a methodology for accurately determining the 3D structure (specifically molecular conformations) at various stages of on-surface reactions. Such information is not easily obtained by scanning probe techniques in the absence of complementary experimental and/or theoretical approaches.

## Methods

### NIXSW and XPS

NIXSW and XPS measurements were performed at Diamond Light Source (I09)^40^ with samples held at room temperature and ultra-high vacuum (UHV); base pressure 4 × 10^−10^ mbar. The I09 beam line consists of two undulator light sources that separately cover “soft” (100–2000 eV) and “hard” (2100–15,000 eV) X-ray energy ranges. The photoelectron spectra were acquired using a VG Scienta EW4000 HAXPES analyser that was mounted perpendicular to the incident light in the same plane as the photon polarisation (linear horizontal). Binding energies defined relative to the Fermi level of the substrate. The reflectivity curves were acquired from a fluorescent plate that was mounted in the port through, which the synchrotron light is incident, and was acquired using a CCD camera mounted on a window opposite that port. The reflectivity curve was fitted to determine the phase of the X-ray standing wave, as well as to model broadening due to experimental uncertainties. Nondipolar effects in the photoelectron yield were modelled using a backward-forward asymmetry parameter, *Q*^41^, derived from the calculations of Nefedov et al.^42^. Due to the large acceptance angle of the EW4000 analyser (~±30°) an average emission angle of 15°, with respect to the surface plane, was used for (111) XSW.

I 3d and C 1s core level XP spectra were obtained using photon energies of 770 eV and 435 eV, respectively. NIXSW measurements were acquired from the (111) and (200) reflection planes (using nominal energies of *E*_Bragg_ = 2629 eV and 3035 eV, respectively) for both I 3d and C 1s. Each NIXSW measurement was repeated at least three times, with each new measurement performed at a different sample location to avoid beam damage. Before and after each measurement, core level spectra for both the I 3d and C 1s were obtained in order to monitor possible beam damage, with no significant changes observed (no observable chemical shift and less than noise level, ~10%, change in peak intensities). Prior to the acquisition of the NIXSW measurement, a reflectivity curve of the respective reflection was acquired in order to judge the quality of that region of the surface, and to ensure that each NIXSW measurement was acquired across the same energy range with respect to the Bragg energy. Averaged spectra for both the I 3d and C 1s measurements were fitted with a convolution of a Doniach–Šunjić line shape^43^ and a Gaussian to obtain intensity information for XSW profiles.

For XSW and XPS experiments a single crystal Ag(111) surface was used, prepared through cycles of Ar ion sputtering (1 keV) for 30 min followed by annealing to 670 K for 30 min. Sample temperatures were estimated based on calibration data obtained from a thermocouple positioned on a dummy sample; the associated uncertainty for temperature measurements is estimated to be ±50 K. TIPB was obtained from Sigma Aldrich and DITP was synthesised using a previously described method^44^. Molecules were deposited at room temperature using a Knudsen cell held at 470 K (TIPB) or 370 K (DITP) for 15 min.

### STM

STM experiments were performed using an Omicron variable temperature (VT) STM system (for the DITP experiments) and an Omicron STM-1 system (for DITP and TIPB experiments) with base pressures of 10^−9^ mbar. Images were acquired at room temperature in constant current mode using electrochemically etched tungsten tips, coated in silver during tip optimisation by controlled indentation into the surface.

STM experiments on both systems used Ag(111) on mica surfaces (Georg Albert PVD GmbH) prepared by cycles of Ar ion sputtering for 30 min at 0.8 keV and 0.75 keV, respectively for 30 min, followed by annealing at 670 K for 30 min. Sample temperatures were estimated using a thermocouple attached to the manipulator arm sample-heating stage and were calibrated based on pyrometer measurements, and thermocouple readings acquired from a dummy sample plate placed in the sample heating stage; the associated uncertainty for temperature measurements is estimated to be ±20 K.

## Supplementary information

Supplementary Information

## Data Availability

The experimental data on which this work is based may be found at 10.17639/nott.7076.

## References

[1] Shen Q, Gao H-Y, Fuchs H (2017). Frontiers of on-surface synthesis: from principles to applications. Nano Today.

[2] Cai J (2010). Atomically precise bottom-up fabrication of graphene nanoribbons. Nature.

[3] Grill L (2007). Nano-architectures by covalent assembly of molecular building blocks. Nat. Nanotechnol..

[4] Lafferentz L (2012). Controlling on-surface polymerization by hierarchical and substrate-directed growth. Nat. Chem..

[5] Bieri M (2010). Two-dimensional polymer formation on surfaces: insight into the roles of precursor mobility and reactivity. J. Am. Chem. Soc..

[6] Blunt MO, Russell JC, Champness NR, Beton PH (2010). Templating molecular adsorption using a covalent organic framework. Chem. Commun..

[7] Lipton-Duffin JA, Ivasenko O, Perepichka DF, Rosei F (2009). Synthesis of polyphenylene molecular wires by surface‐confined polymerization. Small.

[8] Grill L, Hecht S (2020). Covalent on-surface polymerization. Nat. Chem..

[9] Lindner R, Kühnle A (2015). On-surface reactions. ChemPhysChem.

[10] El Garah M, MacLeod JM, Rosei F (2013). Covalently bonded networks through surface-confined polymerization. Surf. Sci..

[11] Dong L, Liu PN, Lin N (2015). Surface-activated coupling reactions confined on a surface. Acc. Chem. Res..

[12] Fan Q, Gottfried JM, Zhu J, Surface-Catalyzed C-C (2015). Covalent coupling strategies toward the synthesis of low-dimensional carbon-based nanostructures. Acc. Chem. Res..

[13] Cai Z, She L, Wu L, Zhong D (2016). On-surface synthesis of linear polyphenyl wires guided by surface steric effect. J. Phys. Chem. C.

[14] Saywell A, Browning AS, Rahe P, Anderson HL, Beton PH (2016). Organisation and ordering of 1D porphyrin polymers synthesised by on-surface glaser coupling. Chem. Commun..

[15] Saywell A (2014). Manipulating the conformation of single organometallic chains on Au(111). J. Phys. Chem. C.

[16] Zhong D (2011). Linear alkane polymerization on a gold surface. Science.

[17] Saywell A, Schwarz J, Hecht S, Grill L (2012). Polymerization on stepped surfaces: alignment of polymers and identification of catalytic sites. Angew. Chem. Int. Ed..

[18] Judd CJ, Haddow SL, Champness NR, Saywell A (2017). Ullmann coupling reactions on Ag(111) and Ag(110); substrate influence on the formation of covalently coupled products and intermediate metal-organic structures. Sci. Rep..

[19] Fan Q (2016). Confined synthesis of organometallic chains and macrocycles by Cu–O surface templating. ACS Nano.

[20] Judd CJ, Champness NR, Saywell A (2017). An on-surface reaction confined within a porous molecular template. Chem. Eur. J..

[21] Judd CJ, Kondratuk DV, Anderson HL, Saywell A (2019). On-surface synthesis within a porphyrin nanoring template. Sci. Rep..

[22] Oteyza DG (2013). Direct imaging of covalent bond structure in single-molecule chemical reactions. Science.

[23] Schuler B (2016). Reversible Bergman cyclization by atomic manipulation. Nat. Chem..

[24] Sweetman A, Champness NR, Saywell A (2020). On-surface chemical reactions characterised by ultra-high resolution scanning probe microscopy. Chem. Soc. Rev..

[25] Schuler B (2013). Adsorption geometry determination of single molecules by atomic force microscopy. Phys. Rev. Lett..

[26] Eichhorn J (2014). On-surface Ullmann polymerization via intermediate organometallic networks on Ag(111). Chem. Commun..

[27] Di Giovannantonio M (2016). Mechanistic picture and kinetic analysis of surface-confined Ullmann polymerization. J. Am. Chem. Soc..

[28] Di Giovannantonio M (2013). Insight into organometallic intermediate and its evolution to covalent bonding in surface-confined Ullmann polymerization. ACS Nano.

[29] Jones RG (2002). X-Ray standing waves at surfaces. J. Phys. Condens. Matter.

[30] Woodruff DP (2005). Surface structure determination using X-ray standing waves. Rep. Prog. Phys..

[31] Fan Q (2018). Surface adatom mediated structural transformation in bromoarene monolayers: precursor phases in surface Ullmann reaction. ACS Nano.

[32] Lackinger M (2017). Surface-assisted Ullmann coupling. Chem. Commun..

[33] Bushell J (2005). The reactive chemisorption of alkyl iodides at Cu(110) and Ag(111) surfaces: a combined STM and XPS study. J. Phys. Chem. B.

[34] Almenningen A (1985). Structure and barrier of internal rotation of biphenyl derivatives in the gaseous state: part 1. The molecular structure and normal coordinate analysis of normal biphenyl and pedeuterated biphenyl. J. Mol. Struct..

[35] Bürker C (2013). Exploring the bonding of large hydrocarbons on noble metals: diindoperylene on Cu(111), Ag(111), and Au(111). Phys. Rev. B.

[36] Björk J (2016). Reaction mechanisms for on-surface synthesis of covalent nanostructures. J. Phys. Condens. Matter.

[37] Wang W, Shi X, Wang S, Van Hove MA, Lin N (2011). Single-molecule resolution of an organometallic intermediate in a surface-supported Ullmann coupling reaction. J. Am. Chem. Soc..

[38] Antes I, Frenking G (1995). Theoretical studies of organometallic compounds. XIV. Structure and bonding of the transition metal methyl and phenyl compounds MCH3 and MC6H5 (M = Cu, Ag, Au) and M(CH3)2 and M(C6H5)2 (M = Zn, Cd, Hg). Organometallics.

[39] Krug CK (2018). Organometallic ring vs. chain formation beyond kinetic control: steering their equilibrium in two-dimensional confinement. Chem. Commun..

[40] Lee T-L, Duncan DA (2018). A two-color beamline for electron spectroscopies at diamond light source. Synchrotron Radiat. News.

[41] Fisher CJ (1998). Non-dipole photoemission effects in x-ray standing wavefield determination of surface structure. J. Phys. Condens. Matter.

[42] Nefedov VI, Yarzhemsky VG, Nefedova IS, Trzhaskovskaya MB, Band IM (2000). The influence of non-dipolar transitions on the angular photoelectron distribution. J. Electron Spectrosc. Relat. Phenom..

[43] Doniach S, Sunjic M (1970). Many-electron singularity in X-ray photoemission and X-ray line spectra from metals. J. Phys. C.

[44] Kokado K, Tokoro Y, Chujo Y (2009). Luminescent and axially chiral π-conjugated polymers linked by carboranes in the main chain. Macromolecules.

