# Supplementary material for: Structural characterisation of molecular conformation and the incorporation of adatoms in an on-surface Ullmann-type reaction
Source: Commun Chem. 2020 Nov 11;3:166. doi: 10.1038/s42004-020-00402-0 (PMC9814584; doi:10.1038/s42004-020-00402-0)
Supplement: Supplementary file 1 — Supplementary Information [file 42004_2020_402_MOESM1_ESM.pdf]

Structural characterisation of molecular conformation and the incorporation of adatoms in an on-surface Ullmann-type reaction

Chris J. Judd[1], Filipe L.Q. Junqueira[1], Sarah L. Haddow[2], Neil R. Champness[2], David A. Duncan[3], Robert G. Jones[2], Alex Saywell[1]*

[1]School of Physics & Astronomy, The University of Nottingham, Nottingham, NG7 2RD, UK.

[2]School of Chemistry, The University of Nottingham, Nottingham, NG7 2RD, UK.

[3]Diamond Light Source, Harwell Science and Innovation Campus, Didcot, OX11 0QX, UK.

*Corresponding author A.S. (alex.saywell@nottingham.ac.uk)

Supporting Information

Contents

Carbon XPS 2
Iodine XPS 3
C 1s NIXSW Analysis 4
I 3d NIXSW Analysis 10
Analysis of (200) reflection C 1s NIXSW measurements 13
Fitting NIXSW Measurements of Organometallic Carbon Atoms 14
STM overview examples 15
Adsorption Modelling Methods 16
TP Lateral Adsorption Modelling 18
References 21

Carbon XPS

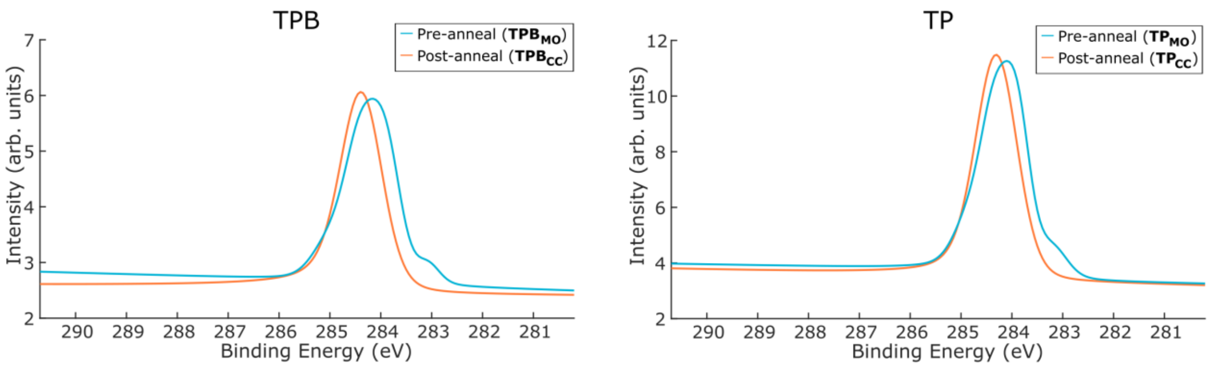

Figure S1. Comparisons of Hard XPS measurements for 1,3,5-triphenylbenzene (TPB), left, and m-terphenyl (TP), right, before and after annealing (blue and orange curves respectively). Spectra calibrated such that peaks positions are at the same binding energy as observed in soft XP spectra.

Figure S1 shows hard X-ray photoelectron spectroscopy (XPS) measurements (incident photons at 2629 eV) of the C 1s region for 1,3,5-triphenylbenzene (TPB) and m-terphenyl (TP) molecules before and after annealing (metal-organic (MO) phase and covalently coupled phase, respectively).

Iodine XPS

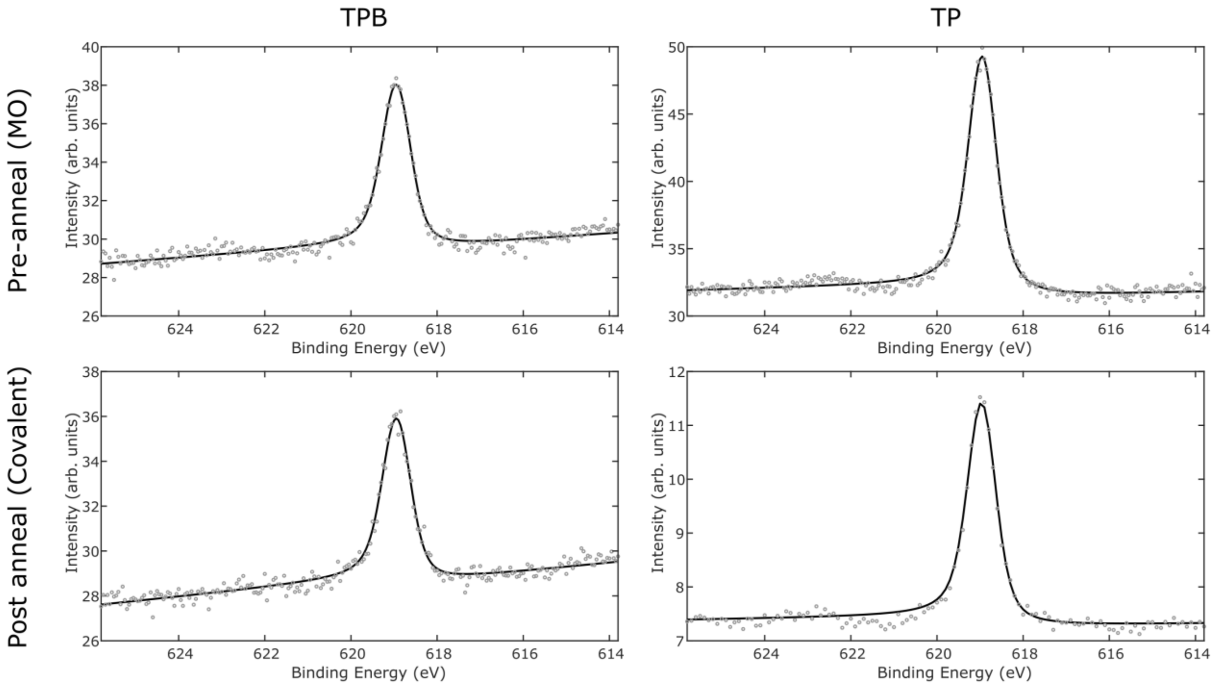

Figure S2. Hard X-ray photoelectron spectra for the I $3d_{5/2}$ region, comparing Ag(111) samples with TPB and TP species, before (MO phase) and after annealing (covalently coupled product). Graphs averaged from 6 scans of the region. Spectra calibrated such that peaks positions are at the same binding energy as observed in soft XP spectra.

Figure S2 shows hard XPS measurements (2629 eV) for TP and TPB, before and after annealing the surface (I $3d_{5/2}$ region). Each graph shows a single peak centred on 619.0 eV, indicating that the iodine moieties on the surface exist in a single chemical state.

C 1s NIXSW Analysis

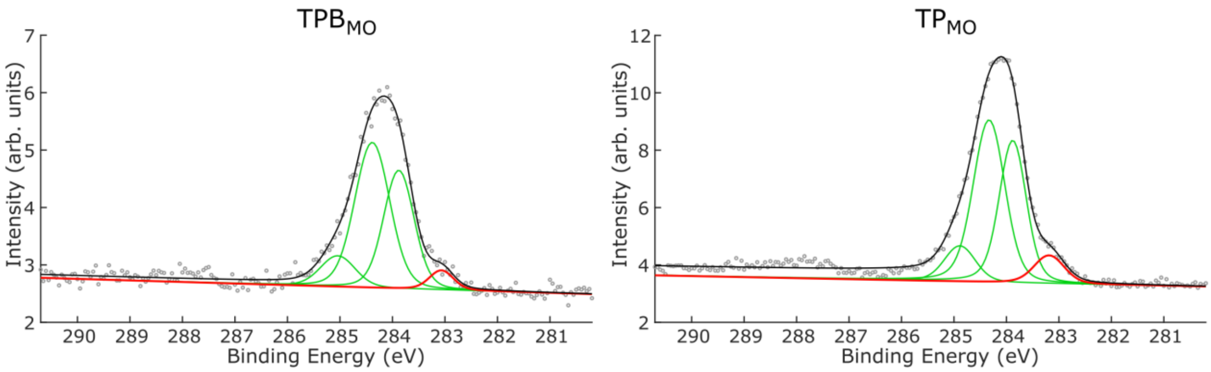

Figure S3. Examples of methods used to fit curves to pre-anneal (MO phase) C 1s data for TPB$_{MO}$ (left) and TP$_{MO}$ (right). Four peaks are used to create the shape of the curve observed in the data. The areas of the three peaks at higher binding energies (green) are summed to extract data for the main peak. The peak at lower binding energy (red) is used to gain information on the shoulder feature. The black line shows the overall fit, summed from all four peaks, to the data, shown in grey.

The three peaks shown in green at higher binding energies (centred on, from left to right, 285.1 eV, 284.4 eV, 283.9 eV for TPB$_{MO}$ and 284.8 eV, 284.3 eV, 283.8 eV for TP$_{MO}$) are summed to obtain the fitting for the main peak, and are attributed to aromatic carbon atoms. Three components were necessary to reproduce the shape of the main peak, and allow the intensity of the shoulder peak due to C-Ag-C to be evaluated. We do not consider the three main peak components to be sufficiently unique or attributable to specific chemical environments within the molecule to allow them to be used for adsorption site. The single peak shown in red (centred on 283.1 for both TPB$_{MO}$ and TP$_{MO}$) is used to extract information from the shoulder feature, attributed to the organometallic carbon species.

Table S1. C 1s (111) XSW data. Data for Coherent fraction (C_f) and position (C_p) values obtained from C 1s (111) reflection NIXSW data and end phenyl group dihedral angle Θ for TP and TPB.

C 1s (111)	Structure Type	Carbon Type	C_f	C_p	Dihedral Angle Θ (°)
TP	MO (TP_{MO})	MO Organometallic	0.79 ± 0.14	0.05 ± 0.06	
	MO (TP_{MO})	MO Aromatic	0.49 ± 0.03	0.28 ± 0.02	29 ± 1
	Covalent (TP_{CC})	Aromatic	0.56 ± 0.03	0.32 ± 0.02	28 ± 1
TPB	MO (TPB_{MO})	MO Organometallic	0.94 ± 0.11	0.03 ± 0.05	
	MO (TPB_{MO})	MO Aromatic	0.50 ± 0.02	0.28 ± 0.01	26 ± 1
	Covalent (TPB_{CC})	Covalent Aromatic	0.43 ± 0.06	0.30 ± 0.04	31 ± 3

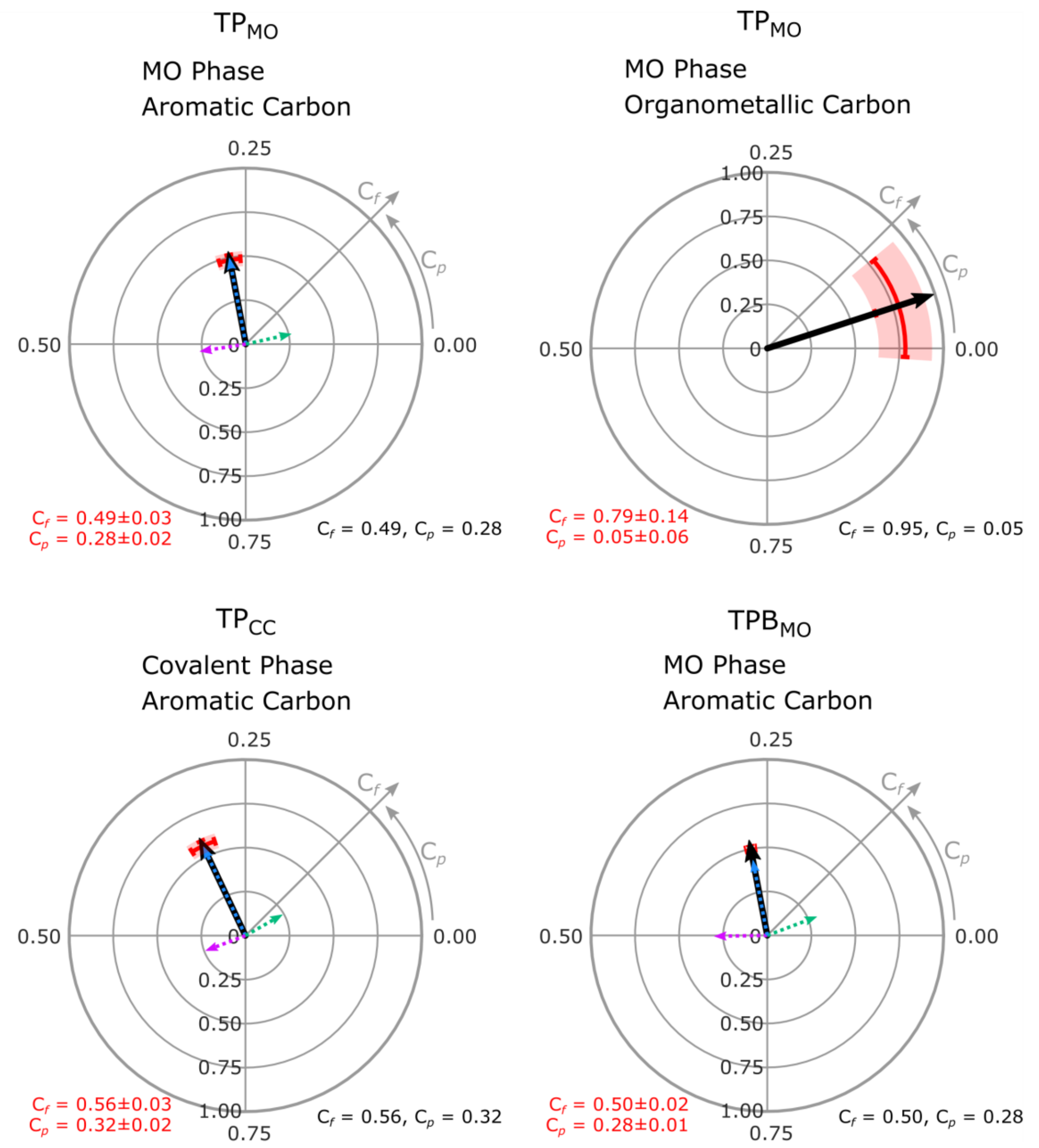

Figure S4. Additional Argand diagrams showing coherent fraction and position values obtained from XSW measurements from the (111) reflection. Shown are the measurements of aromatic (top left) and organometallic (top right) carbons for TP in the MO phase, as well as measurements of the covalent phase aromatic carbons for TP_{CC} (bottom left) and TPB MO phase (bottom right). Calculated values for C_f and C_p based upon the model for twisted phenyl rings (with Θ for each model given in Table S1 - see discussion in main manuscript), are presented. Each arrow is a vector showing C_f and C_p values in polar co-ordinates. Dashed arrows represent aromatic carbon atoms higher (purple), lower (green) and level with the molecular plane (blue). The solid black arrow shows the predicted C_f and C_p values for our model and is in excellent agreement with experimental values (red region indicates data point and estimate of corresponding error).

Model for obtaining the dihedral angle (Θ)

In order to determine the dihedral angle, Θ, for the rotated side groups in TP and TPB, a simplified model was developed based upon the NIXSW data (as employed in the 'Molecular adsorption and conformation: chemically sensitive NIXSW analysis' section of the main manuscript). The geometric model is shown in Figure S5. Within this model, the central aryl groups are flat and planar relative to the surface plane resting at a single distance *d* from the closest reflecting plane; this is defined as the molecular plane. The central aryl groups are connected by side aryl groups by C-C bonds that are colinear and lie within the molecular plane. The side aryl groups are twisted by an angle Θ about these bonds (orange dot in Fig S5 inset) causing two side aryl carbon atoms to be displaced upwards, and two downwards, by d_{twist}.

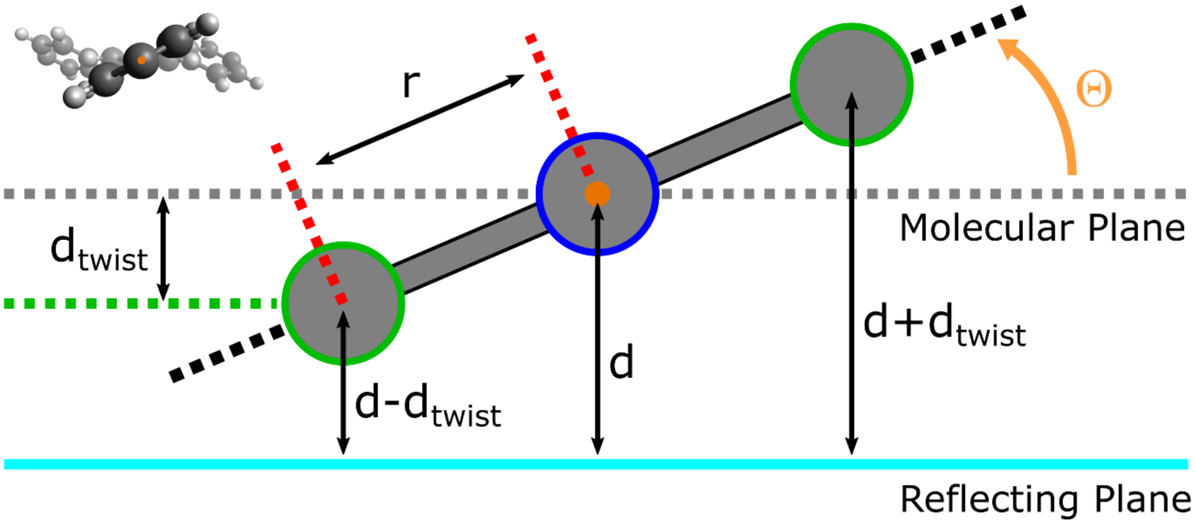

Figure S5. Model for calculating twisting angle for TP and TPB using NIXSW measurements. Average molecule height is shown as the molecular plane a distance *d* above the closest reflecting plane. Grey circles represent carbon atoms as part of the aryl side groups of TP/TPB, with blue highlighted atoms in line with the molecular plane and green highlighted atoms twisted by angle Θ such that they are offset from the molecular plane by distance d_{twist}. Green carbon atoms are a distance *r* from the axis of rotation (orange dot). Inset (top left) shows an example TPB unit with twisted side aryl groups as in this model.

The total coherent fraction, C_f, and coherent position, C_p, for a system with n different positions, each with a coherent fraction and position (f_n and p_n), is defined by[1]

$$C_f e^{2\pi i C_p} = \sum_n f_n e^{2\pi i p_n}. \tag{1}$$

Carbon atoms within this model can be separated into two types: either remaining fixed (*fixed*) at the molecular plane or located above or below due to twisting (*twist*). The fractions, f, for each of these atoms are given by

$$f_{fixed} + f_{twist} = 1. \tag{2}$$

The coherent position for the fixed atoms, which due to symmetry is equal to the total coherent fraction, is given by

$$p_{fixed} = \frac{d}{D} = C_p \tag{3}$$

where D is the separation of the reflecting planes [this holds for $d \leq D$]. Similarly the coherent position for the displaced carbon atoms can be written as

$$p_{twist} = p_{fixed} \pm \frac{d_{twist}}{D} = p_{fixed} \pm \frac{r \sin \Theta}{D} \tag{4}$$

with r being the separation between a twisted atom and the axis of rotation.

Applying the details of the twisting model shown above to equation (1), C_f and C_p values can be calculated such that

$$\begin{aligned} C_f e^{2\pi i C_p} = {} & f_{fixed} e^{2\pi i p_{fixed}} \\ & + \frac{1}{2} f_{twist} e^{2\pi i \left(p_{fixed} + \frac{r \sin \Theta}{D}\right)} \\ & + \frac{1}{2} f_{twist} e^{2\pi i \left(p_{fixed} - \frac{r \sin \Theta}{D}\right)}. \end{aligned} \tag{5}$$

Utilising the fact that the total coherent position is equivalent to the coherent position of fixed carbon atoms (equation (3)), equation 4 simplifies to

$$C_f = f_{fixed} + f_{twist} \cos\left(\frac{2\pi r \sin\Theta}{D}\right). \tag{6}$$

Finally, using equation 2 yields

$$C_f = f_{fixed} + (1 - f_{fixed}) \cos\left(\frac{2\pi r \sin\Theta}{D}\right) \tag{7}$$

C_f was determined experimentally from NIXSW measurements and all other values are known. Therefore, using equation 7, values for Θ could be determined for both TP and TPB in MO and covalent phases of the Ullmann reaction. r was determined to be 0.1205 nm, based on molecular mechanics simulations of TPB and TP. f_{fixed} values were determined from the number of carbon atoms at each position (e.g. for TP_{MO} and TP_{CC}, f_{fixed}= 10/18, f_{twist}=8/18. For TPB_{MO} and TPB_{CC}, f_{fixed}=12/24 and f_{twist}=12/24) with final values multiplied by 0.95 to account for small fluctuations in atomic positions caused by effects such as thermal vibration. Figure S6 shows equation (7) for TPB_{cc} where f_{fixed}=0.5, r=0.1205 nm, and D=0.2361 Å. For $\theta \approx 30^\circ$, which matches the experimental C_f value, an error of $\pm 3^\circ$ corresponds to ± 0.07 in C_f.

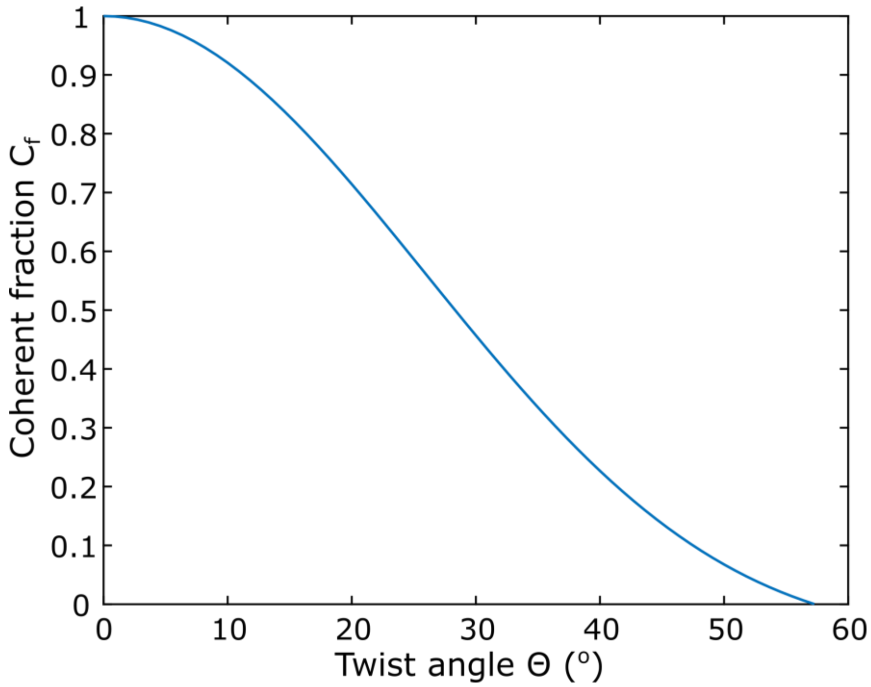

Figure S6. Variation of coherent fraction C_f with twist angle Θ for TPB_{CC} obtained from equation (7).

I 3d NIXSW Analysis

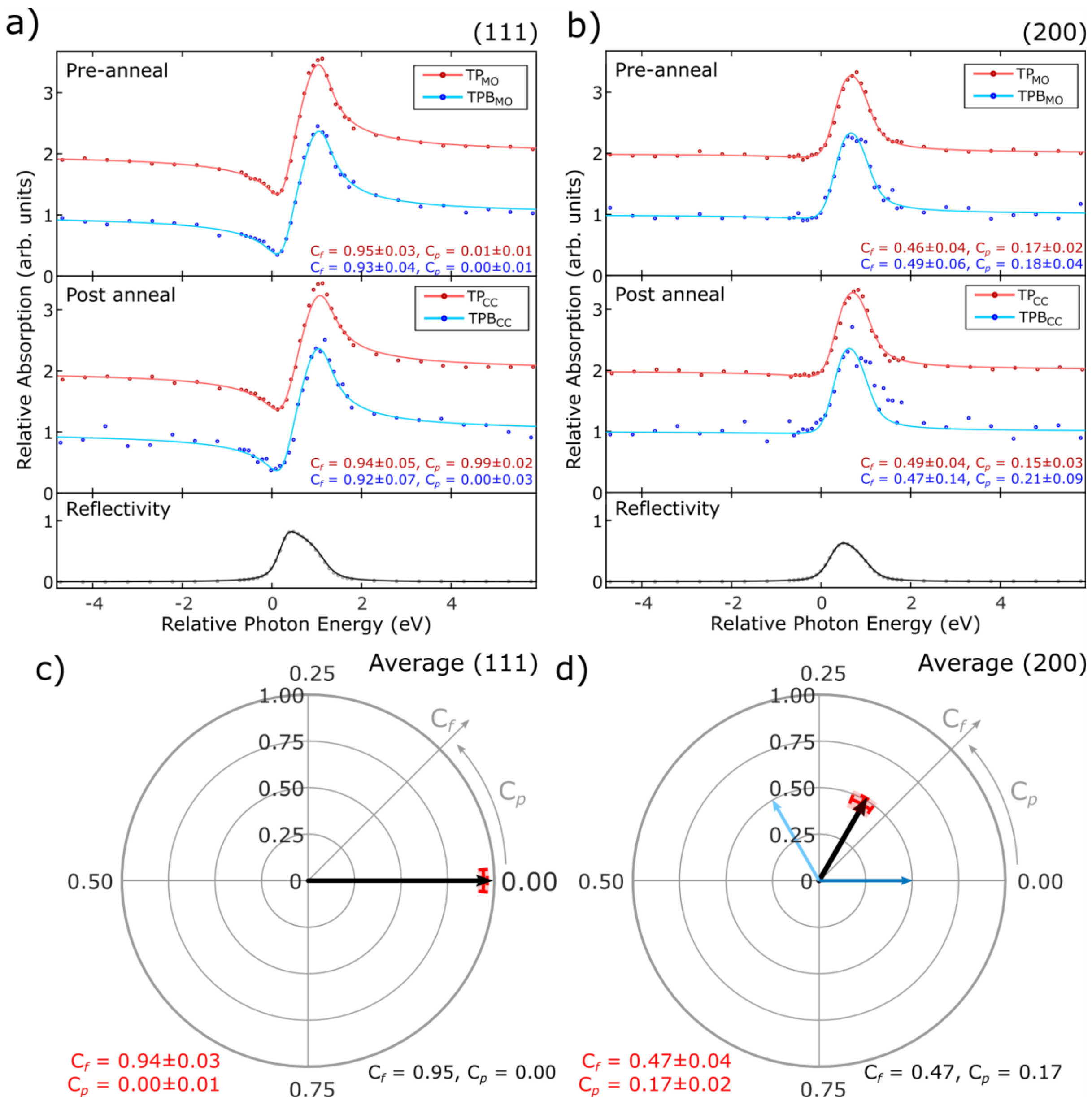

Figure S7. NIXSW characterisation of the iodine species (I 3d core level) present as part of TP and TPB assemblies on Ag(111) in the metal-organic structure (pre-anneal) and the covalently bonded structure (post-anneal). [(a) and (b) NIXSW photoemission yields obtained using the (111) and (200) reflections, respectively, comparing TP (red) and TPB (blue) before and after annealing]. Each profile is normalised to a value of 1 away from the Bragg condition, with TP results offset by 1 unit for clarity. The obtained coherent fraction (C_f) and position (C_p) values are shown for each profile. (c) and (d) Argand diagrams showing average coherent fraction, C_f, and, C_p, values for the (111) and (200) reflections. (d) Shows a model consisting of iodine atoms at a position of 2.36 Å (C_p=0.00) above the silver surface (111) plane adsorbed in a 1:1 ratio at hcp:fcc hollow sites, shown as blue arrows (dark blue arrow is the fcc site and the light blue the hcp site). Black arrow shows resultant vector for our proposed models for

iodine, and red error bars show average of all experimental measurements, with C_f and C_p values shown. [NB (111) layer spacing is 2.36Å and the layer spacing of (200) is 2.05 Å]

Table S2 – I 3d XSW data. Coherent fraction (C_f) and position (C_p) values for NIXSW measurements acquired for the I 3d level of TP and TPB on the Ag(111) surface. Acquired for the (111) and (200) reflections.

I3d		(111)		(200)	
		C_f	C_p	C_f	C_p
TP	Pre Anneal	0.95 ± 0.03	0.01 ± 0.01	0.46 ± 0.04	0.17 ± 0.02
	Post Anneal	0.94 ± 0.05	0.99 ± 0.02*	0.49 ± 0.04	0.15 ± 0.03
TPB	Pre Anneal	0.93 ± 0.04	0.00 ± 0.01	0.49 ± 0.06	0.18 ± 0.04
	Post Anneal	0.92 ± 0.07	0.00 ± 0.03	0.47 ± 0.14	0.21 ± 0.09
Average		0.94 ± 0.02	0.00 ± 0.01	0.47 ± 0.04	0.17 ± 0.02

* NB C_p are cyclic such that 0 and 1 are the same.

As the C_f and C_p values from the iodine species are the same, within error, and do not vary significantly with respect to precursor molecule type and annealing we therefore consider an average value in the following discussion. The average of TP and TPB measurements, before and after anneal give a coherent fraction value of $C_{f(111)\text{-}I3d} = 0.94 \pm 0.02$. This high coherent fraction value suggests that all iodine atoms are in similar adsorption sites relative to the (111) Bragg reflection, meaning a single height above the Ag(111) surface. From this and averaged coherent position, $C_{p(111)\text{-}I3d} = 0.00 \pm 0.01$, all iodine atoms are expected to rest in plane with the (111) layer spacing, most likely at the first Ag(111) layer spacing above the surface, at 0.236nm (compatible with a vdW radius of 0.198 nm for Ag). This adsorption model is presented as an Argand diagram in Figure S5c (black arrow), with the NIXSW measured coherent fraction and position values (red error bars).

The precise adsorption site (in 3D) of iodine on the surface can then be determined using NIXSW measurements from the (200) reflection for triangulation.[1,2] Averaging all measurements from the (200) reflection gives coherent fraction $C_{f(200)\text{-}I3d} = 0.47 \pm 0.04$ and position $C_{p(200)\text{-}I3d} = 0.17 \pm 0.02$ (similar for both molecules before and after annealing). The

coherent fraction value of ~0.5 suggests multiple iodine adsorption sites (at least two distinct sites) on the surface. Using the height of iodine above the surface determined from the (111) reflection, a model of iodine adsorbed to HCP and FCC hollow sites in a 1:1 ratio is proposed in Figure S5d. The $C_{f(200)\text{-}I3d}$ and $C_{p(200)\text{-}I3d}$ values for hollow sites are shown as blue arrows with the resultant vector shown in black. This model gives coherent fraction and position values of $C_{f(200)\text{-}I3d} = 0.47$ and $C_{p(200)\text{-}I3d} = 0.17$, in excellent agreement with measured values and is also in agreement with our STM measured adsorption structures for related iodine containing molecules.[3] In addition, these results are in agreement with surface-extended X-ray absorption fine structure (SEXAFS) studies of iodine on Ag(111), where iodine atom adsorption sites are assigned as 3-fold hollow sites and the I-Ag bond length is determined to be 2.87±0.03 Å.[4] The SEXAFS determined bond length corresponds to a height of 2.34±0.04 Å in a 3-fold hollow (relative to the (111) plane) and hence agrees within error of our XSW determined adsorption height of 2.36 Å (with the assumption that there is no relaxation of the top layer of silver atoms).

Analysis of (200) reflection C 1s NIXSW measurements

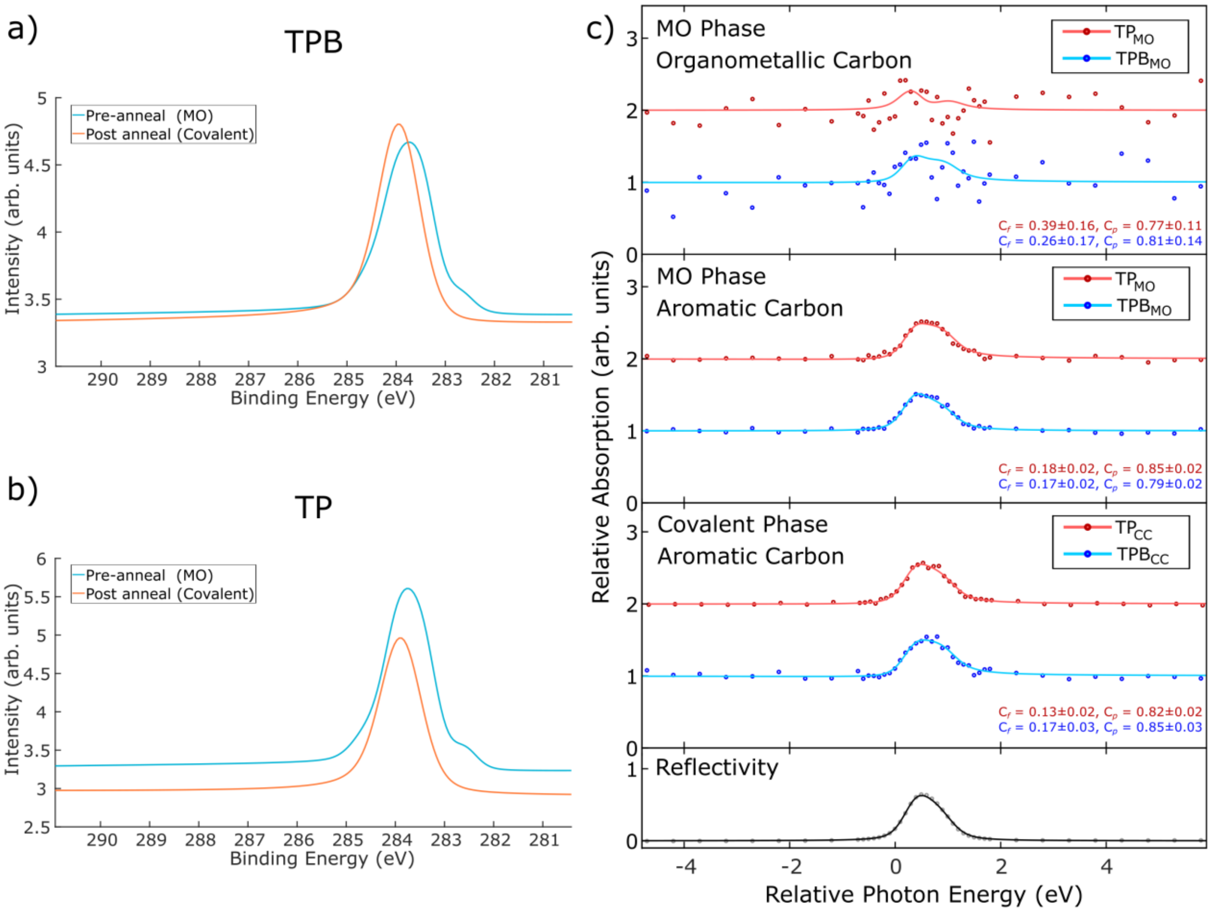

Figure S8. Comparisons of Hard XPS measurements of the C 1s region for TPB (a) and TP (b) in MO and covalent phases (blue and orange curves respectively). (c) NIXSW photoemission yields obtained using the (200) reflection for the C 1s core level, comparing TP (red) and TPB (blue) in MO and covalent phases. Each profile is normalised to 1, away from the Bragg condition, with TP results offset by 1 unit for clarity. The obtained coherent fraction (C_f) and position (C_p) values are shown for each profile.

Table S3. Data for Coherent fraction (C_f) and position (C_p) values obtained from C 1s (200) reflection NIXSW data.

C 1s (200)	Phase / Carbon Yype	C_f	C_p
TP	MO / Organometallic	0.39 ± 0.16	0.77 ± 0.11
	MO / Aromatic	0.18 ± 0.02	0.85 ± 0.02
	Covalent / Aromatic	0.13 ± 0.02	0.82 ± 0.02
TPB	MO / Organometallic	0.26 ± 0.17	0.81 ± 0.14
	MO / Aromatic	0.17 ± 0.02	0.79 ± 0.02
	Covalent / Aromatic	0.17 ± 0.03	0.85 ± 0.03

Fitting NIXSW Measurements of Organometallic Carbon Atoms

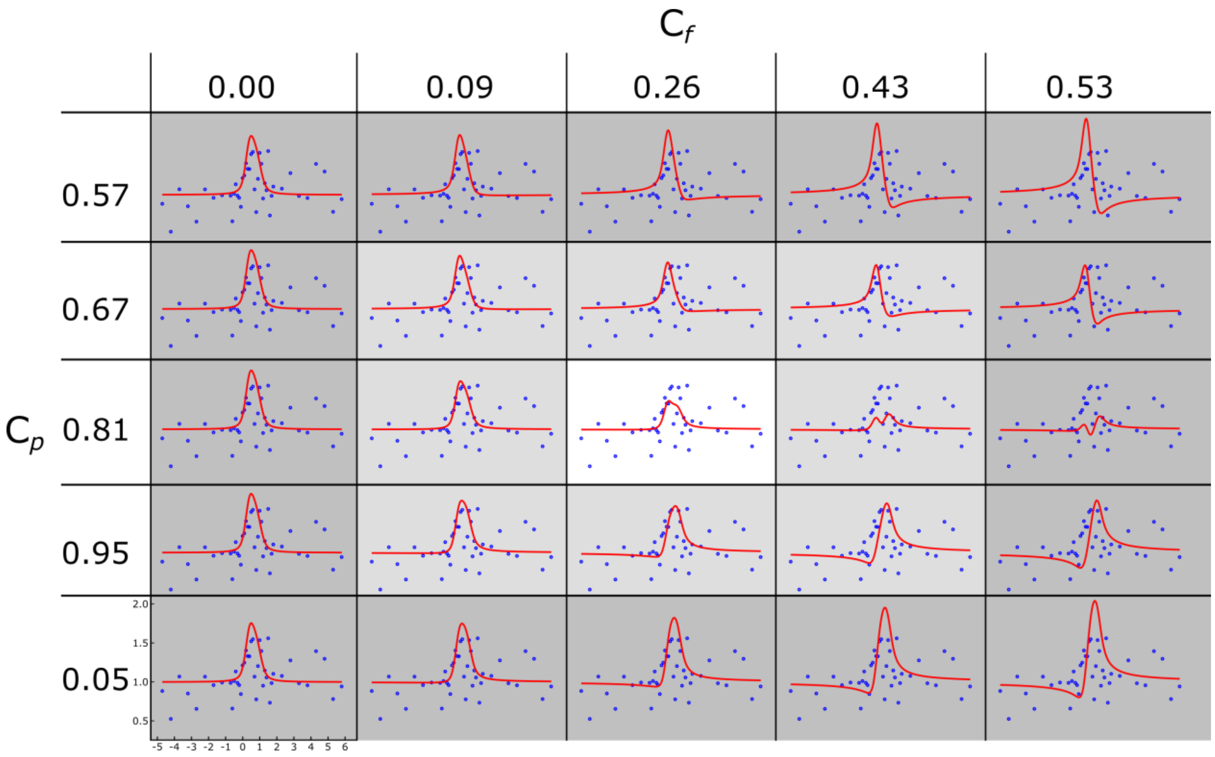

Figure S9. Table of comparisons of different XSW profiles (red) with varied coherent fraction (C_f) and position (C_p) values. Data points (blue) are for TPB measurements of the organometallic carbon atoms from the (200) reflection. Each XSW profile is generated from C_f and C_p values either at the quoted values (white, centre), within the quoted uncertainty (light grey) or 0.1 outside the uncertainty range (dark grey, outer). XSW profile in bottom left shows scale for Relative Absorption (y-axis) and Energy, eV, relative to the Bragg condition (x-axis). Profiles with C_f and C_p values within the uncertainty range are found to acceptably fit the data whereas profiles with values outside this range do not.

Experimentally obtained XSW curves cover a very large range of adsorption versus photon energy. This means that although the data for the organometallic species presented here have a less than ideal signal-to-noise ratio, a very large proportion of C_p & C_f combinations are completely excluded as fits to them, leaving the restricted range of fitting values shown in Fig. S7.

STM overview examples

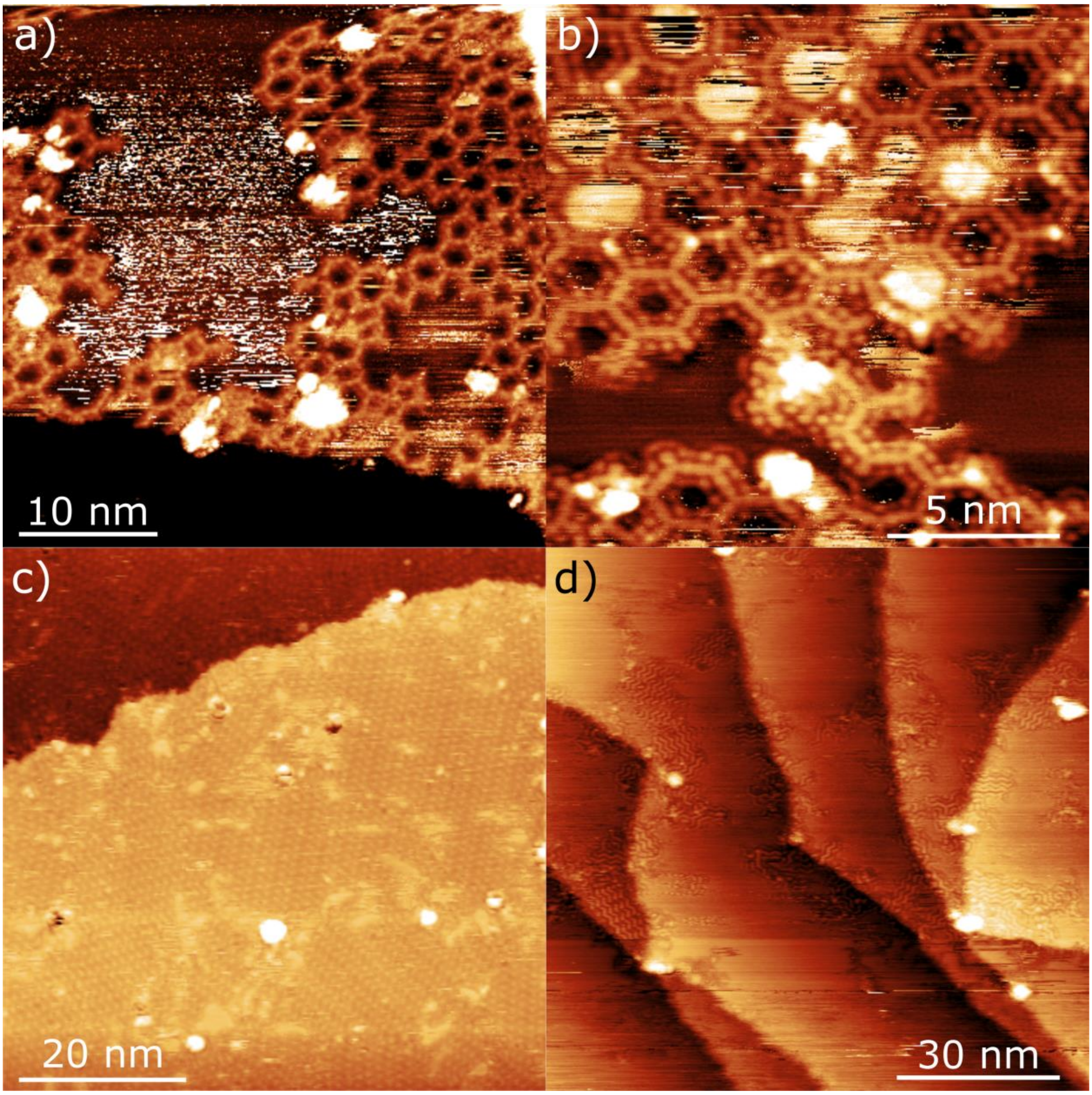

Figure S10. Overview STM images for TPB and TP in MO and covalent bonding phases. (a) MO phase of TPB (TPB_{MO}). Image parameters: $V_{bias} = -1.8$ V $I_{set} = 20$ pA. (b) Covalent phase of TPB (TPB_{CC}). Image parameters: $V_{bias} = -1.8$ V $I_{set} = 20$ pA. (c) MO phase of TP (TP_{MO}). Image parameters: $V_{bias} = 1.5$ V $I_{set} = 5.0$ pA. (d) Covalent phase of TP (TP_{CC}). Image parameters: $V_{bias} = -1.0$ V $I_{set} = 5.0$ pA.

Adsorption Modelling Methods

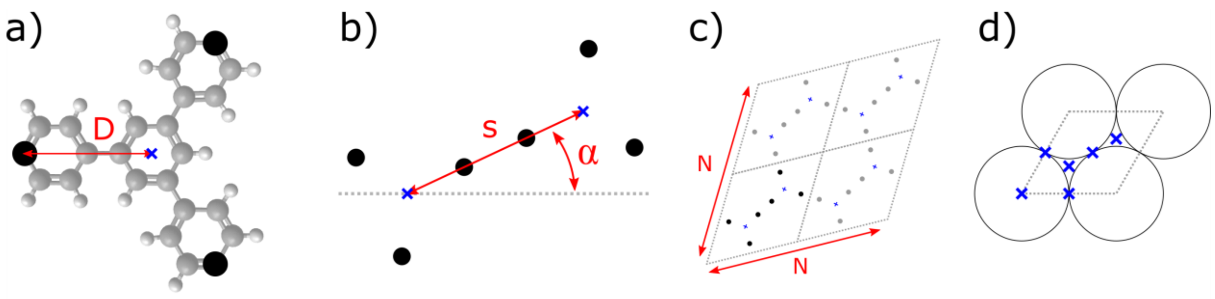

Figure S11. Figures showing methods used to model lateral adsorption of molecules from XSW measurements of organometallic carbon atoms. a) Only organometallic carbon atoms are used within the model, with the aromatic carbon atoms adsorption sites inferred from these positions. The distance, *D*, of these atoms to the centre of the molecule (blue cross) is varied to account for variations in bending of molecule arms down towards the surface. b) An additional molecule is added, rotated 180°, to form a molecular unit cell and the separation of the two molecules, *s*, and their angle relative to the x axis ($[1\bar{1}0]$ direction), α, is allowed to vary. c) The unit cell is copied N times in two directions to account for extended structures on the surface. d) Positions of molecules were allowed to vary laterally relative to the surface. Only models where all molecules have their centres in a high symmetry (atop, bridge or three-fold hollow – shown as blue crosses) site are considered for simplicity.

The method used for building a model of molecules is described in Figure S9. Firstly, (a) shows the simplified model of TPB molecules used. Only organometallic carbon atoms (black circles) were considered as only these atoms contribute to the organometallic carbon features seen in XSW measurements. The distance, *D*, between the carbon atoms and the centre of the molecule (blue cross) can be varied by small amounts to account for stretching of the molecule and the end phenyl groups bending down towards the surface (from (111) XSW results). Next, an additional molecule is added (rotated by 180°) to create the molecular overlayer structure observed in STM images. The separation of molecule centres, s, as well as the unit cells rotation relative to the x-axis, α, can be varied (Figure S9b) to account for different positioning of molecules on the surface. Then, the unit cell is repeated N = 6 times to create an extended structure (Figure S9c). This accounts for the fact that the molecular adlayer has a regular

periodicity but is not necessarily coincident with the underlying Ag(111) unit mesh. Finally, the model is placed over a Ag(111) surface model, at a height above the surface determined from (111) XSW measurements of organometallic carbons. The model assumes the central phenyl ring of all molecules will be adsorbed at a high symmetry site (atop, bridge or three-fold hollow) on the surface (Figure S9d). This was done to simplify calculations as otherwise there would be a near infinite number of sites that would satisfy C_f and C_p values. In addition to this, the model was extended to include ±120° rotations and the reflection over the $[1\bar{1}0]$ direction to account for additional domains that form due to the symmetry of the (111) crystal surface.

Using this model and adjusting its variables (D, s, α, height and molecule centre lateral position relative to the surface), the distances of all organometallic carbon atoms from the (200) reflecting plane can be calculated and used to determine an expected overall C_f and C_p values for the model.[1] D was taken to be 0.56 ± 0.02 nm (based upon dimensions of the gas phase molecule determined via a molecular mechanics-based model), the other variables are based upon the STM measured dimensions and those acquired from the (111) reflection XSW data.

These calculated values of coherent fraction and position were then compared to the measured C_f and C_p values to determine which models produced values that were within the range of uncertainties. This methodology was used to model both TPB and TP structures, based on XSW and STM data, reported upon in the main manuscript.

TP Lateral Adsorption Modelling

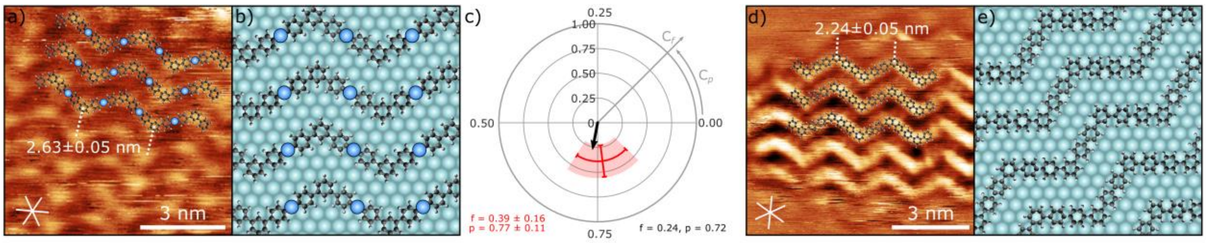

Figure S12. Figure detailing adsorption of DITP on Ag(111). (a) STM image of TP in the metal-organic phase (TP_{MO}) of the Ullmann coupling reaction. TP units and silver adatoms are overlaid to show structures formed with molecule-molecule separation and surface lattice directions indicated (white - bottom left). Image parameters: V_{bias} = 1.8 V I_{set} = 20.0 pA. (b) Proposed adsorption model for metal-organic phase of TP_{MO} on Ag(111) based on XSW modelling. (c) Argand diagram for TP_{MO}, showing resultant vector (black) for organometallic carbon atom locations relative to the (200) plane, based on proposed adsorption model (b). Measured C_f and C_p values are shown in red and the model C_f and C_p values are given in the bottom right. (d) STM image of covalently coupled TP (TP_{CC}), with molecule-molecule separation and surface lattice (white - bottom left) shown. Image parameters: V_{bias} = -1.8 V I_{set} = 10.0 pA. (e) Proposed adsorption model for covalent phase of TP (TP_{CC}). Model based on measurements obtained from STM images.

Using a similar method to that used for TPB, the lateral adsorption geometry of TP on Ag(111) was investigated by combining NIXSW and STM measurements. Room temperature STM images of TP on Ag(111), in both the metal-organic and covalently coupled phases, were obtained previously.[5] An example STM image of the metal-organic phase is shown in Figure S10a. TP_{MO} units appear as periodic circular bright features, arranged in islands of parallel zigzag chains. Islands were observed in three different rotations, matching the symmetries of the surface. Topographic features associated with Ag adatoms are not seen in STM images (noted previously[6]). Instead, their positions are inferred from the arrangement of TP_{MO} units and a model is overlaid to show this. Measurements from STM images show the zigzag chains to have a period of 2.63 ± 0.05 nm, and are oriented at approximately -5° relative to the $\langle\bar{1}10\rangle$ set of crystal directions. Zigzag chains were found to be separated by 1.14 ± 0.05 nm.

From measurements obtained from STM images, a simplified model was developed to simulate how structures adsorb relative to the surface. The model considers only organometallic carbon atoms for simplicity, with the rest of the molecule inferred from their positions. Variables within the model structure included molecular separation, chain separation, angle relative to the surface, height above the surface and lateral position of molecules (See Adsorption Modelling Methods section below for further details).

The variables were systematically altered within a small range to find surface geometries with calculated C_f and C_p values that agreed with XSW measurements. For TP_{MO}, only a single physically reasonable structural arrangement (with all Ag adatoms positioned above high symmetry sites) was found that agreed with both STM and XSW measurements. Slight variations in adsorption geometries were possible for this molecular structure through small variances in adsorption height and the distances of carbon atoms to molecular centres. An example model is shown in Figure S10b. Molecule centres are adsorbed at similar bridge sites, with molecules in adjacent chains adsorbed at a different bridge site. Ag adatoms are found to alternate between adsorbing at bridge and atop sites and only bridge sites for adjacent chains.

The adsorption model shows zigzag chains to have a period 2.60 nm, at an angle of 0° relative to the $<\bar{1}10>$ set of crystal directions, in agreement with STM measurements (2.63 ± 0.05 nm and -5° respectively). Additionally, the adsorption model has molecular chains separated by 1.13 nm which is in good agreement with the measured value of 1.14 ± 0.05 nm.

An argand diagram showing the resultant C_f and C_p values for this model is shown in Figure S10c. For metal-organic TP, all valid models gave values of $C_{f(200)\text{-}C1s} = 0.24 \pm 0.01$ and $C_{p(200)\text{-}C1s} = 0.72 \pm 0.03$. Similar to models of TPB_{MO}, both C_f and C_p values are lower than expected from measurements ($C_{f(200)\text{-}C1s} = 0.39 \pm 0.16$, $C_{p(200)\text{-}C1s} = 0.77 \pm 0.11$) but still agree within our experimental uncertainty.

STM experiments were also performed for the covalent phase of TP (TP_{CC}) and an example STM image of an island of molecules is shown in Figure S10d. Zigzag chains have constant apparent height along their lengths, indicating that molecules are covalently bonded. A molecular structure for the zigzag chains is overlaid. TP_{CC} molecular chains were found to have a period of 2.24 ± 0.05 nm at an angle of 31° to the underlying surface. Chains were found to be separated by 1.19 ± 0.05 nm.

As with TPB_{CC}, simulations using NIXSW measurements of C_f and C_p values were not used to develop adsorption models of covalent TP_{CC}, due to the complexity of molecules and the number of possible variables within the system. Instead, adsorption models were developed based on measured dimensions and angles relative to the surface, attempting to align molecule centres to high symmetry sites. A tentative adsorption model developed using these methods is shown in Figure S10e. Chains in this model have a period of 2.25 nm, are oriented at 30° to the underlying surface and are separated by 1.15 nm.

References

(1) Woodruff, D. P. Surface Structure Determination Using X-Ray Standing Waves. *Rep. Prog. Phys.* **2005**, *68* (4), 743–798. https://doi.org/10.1088/0034-4885/68/4/R01.

(2) Jones, R. G.; Chan, A. S. Y.; Roper, M. G.; Skegg, M. P.; Shuttleworth, I. G.; Fisher, C. J.; Jackson, G. J.; Lee, J. J.; Woodruff, D. P.; Singh, N. K.; Cowie, B. C. C. X-Ray Standing Waves at Surfaces. *J. Phys. Condens. Matter* **2002**, *14* (16), 4059–4074. https://doi.org/10.1088/0953-8984/14/16/301.

(3) Saywell, A.; Greń, W.; Franc, G.; Gourdon, A.; Bouju, X.; Grill, L. Manipulating the Conformation of Single Organometallic Chains on Au(111). *J. Phys. Chem. C* **2014**, *118* (3), 1719–1728. https://doi.org/10.1021/jp409323g.

(4) Citrin, P. H.; Eisenberger, P.; Hewitt, R. C. Extended X-Ray-Absorption Fine Structure of Surface Atoms on Single-Crystal Substrates: Iodine Adsorbed on Ag(111). *Phys. Rev. Lett.* **1978**, *41* (8), 598–598. https://doi.org/10.1103/PhysRevLett.41.598.3.

(5) Judd, C. J.; Haddow, S. L.; Champness, N. R.; Saywell, A. Ullmann Coupling Reactions on Ag(111) and Ag(110); Substrate Influence on the Formation of Covalently Coupled Products and Intermediate Metal-Organic Structures. *Sci. Rep.* **2017**, *7* (1), 14541. https://doi.org/10.1038/s41598-017-13315-1.

(6) Gutzler, R.; Walch, H.; Eder, G.; Kloft, S.; Heckl, W. M.; Lackinger, M. Surface Mediated Synthesis of 2D Covalent Organic Frameworks: 1,3,5-Tris(4-Bromophenyl)Benzene on Graphite(001), Cu(111), and Ag(110). *Chem Commun* **2009**, *0* (29), 4456–4458. https://doi.org/10.1039/B906836H.
